# Report on Materia Medica and Therapeutics

**Published:** 1858-09

**Authors:** John Bell


### REPORT ON MATERIA MEDICA AND THERAPEUTICS FOR THE YEAR 1857.

By JOHN BELL, M.D.

### PART I.

A REPORT on American contributions to Materia Medica and Therapeutics for the year 1857, may, appropriately enough, be preceded by a brief notice of the labors of American writers in these departments.

The first attempt towards the formation of an American Materia Medica, by an investigation of the medicinal properties of our indigenous plants, was made by a learned German, Dr. Shœpf, who visited this country about the year 1786. In 1787 he published the result of his labors in a small volume, entitled *Materia Medica Potissimum Regni Vegetabilis.* This work was followed by a paper which appeared in the *Amœnitates Academicæ,* (Vol. IV. Dissert, LXXII. p. 522,) under the title of *Specifica Canadensium,* in which Koel, the author, enumerates and describes a few indigenous medicinal plants. Among our home contributions in this line, priority must be given to a paper read before the Philadelphia Medical Society, bearing the title of "Collections for an Essay toward a Materia Medica of the United States," by Dr. Benjamin Smith Barton. A second edition of this address appeared in the latter part of the year 1801, with the addition of notes and an appendix, and a third in 1810, a copy of which is now on our table. In this last is included a second part, which bears the date of 1804. Dr. Barton early announced his intention "to publish a series of engravings of the principal medicinal vegetables of our country," in a quarto form, nearly of the size of the plates in Dr. Woodville's Medical Botany. This design was never carried into practice by its author, and the execution of it was left for his nephew, Dr. Wm. P. C. Barton. Much zeal was manifested by several of the alumni of the medical department of the University of Pennsylvania, in the closing years of the last, and the first five years of the present century, in their investigations into the properties and therapeutical value of different indigenous plants. The results were, for the most part, given in the inaugural theses of the several experimenters, who had been invited to undertake what they did, mainly by the promptings and encouragement of Dr. B. S. Barton, then Professor of Materia Medica and Botany in the University.

The curiosity of our readers will, perhaps, prompt them to ask for the names of the indigenous plants now referred to, but without their caring for repetition, in each case, of the formal title of the inaugural essay. Under this supposition, we give the following list of the subjects of the essays, and the names of their authors:—1. *Cornus Florida,* 1802; 2. *Kalmia Latifolia* and *Angustifolia,* 1802; 3. *Laurus,* a native species of, 1804; 4. *Liriodendron Tulipfera,* or Poplar Tree, 1802; 5. *Lupulus Communis,* or Common Hop, 1805; 6. *Magnolia*

*Tripetalia* and *Magnolia Acuminata*, 1806; 7. *Magnolia Glauca*, or Common White Laurel Tree, 1802; 8. *Persimmon*, 1792; 9. *Phytollacca Decandra*, (Poke Weed,) 1795; 10. *Prunus Virginiana*, or Wild Cherry Tree, 1802; 11. *Arbutus Uva Ursi*, *Pyrola Umbellata* and *Maculata*, 1803; 12. *Rhus vernix*, *Rhus radicans*, *Rhus glabrum*, or Poison ash, Poison vine and common Sumach, 1798; 13. *Sanguinaria Canadensis*, or Puccoon, 1803; 14. *Polygala Senega*, 1803; 15. *Spigelia Marilandica*, or Indian Pink, 1802; 16. *Datura Stramonium*, or common Thorn Apple; 17. *Nicotiana Tabacum*, or common Tobacco; 18. Effects of Tobacco Fumes on the System, and their Use in cases of Suspended Animation from Submersion, 1805. The authors of the above essays are respectively—1. John M. Walker; 2. George G. Thomas; 3. Austin Brocker; 4. Patrick Kerr Rogers; 5. Wakeman Bryarly; 6. John Floyd; 7. Thomas D. Rose; 8. James Woodhouse; 9. Benjamin Shultze; 10. Charles Morris; 11. S. Mitchell; 12. Thomas Horsfield; 13. William Downey; 14. Thomas Massie; 15. Hedge Thompson; 16. Samuel Cooper; and 17. Edward Bradford.

In the same year (1817) there were begun to be published two works, in numbers, illustrating indigenous Materia Medica; the one by Dr. Wm. P. C. Barton, of Philadelphia, the other by Dr. Jacob Bigelow, of Boston. The former was called "Vegetable Materia Medica of the United States; or Medical Botany." The work consists of two volumes, quarto, with sixty plates of colored engravings. Dr. Bigelow's work is in three volumes, large octavo, and gives the colored engravings of sixty plants. It is entitled "Medical Botany, being a collection of the native and medicinal plants of the United States." Rafinesque's Medical Flora made its appearance about this time. The next work, illustrative of Medical Botany of a later date, which includes both indigenous and exotic plants, is that by Dr. Joseph Carson. Its author declares his object in preparing it, is to present the botanical history of the Materia Medica. All therapeutical and pharmacological details have been omitted. The work appears in five volumes, quarto, with a hundred colored illustrations. Dr. R. E. Griffith is the author of a volume on Medical Botany, illustrated with wood-cuts. Of anterior date to any of the works now mentioned, was Thacher's New American Dispensatory, 1810. To the labors of Dr. Ives, of Yale College, is the country indebted for improvement of our indigenous Materia Medica. Dr. Tully has been an industrious worker in the same field, as we see by his essays on the *Sanguinaria Canadensis* and *Scutellaria Lateriflora*, and since in his systematic treatise on Materia Medica, or Pharmacology and Therapeutics, now in course of publication, in numbers. Much useful information on the curative powers of indigenous plants of the United States will be found in the pages of the "New York Medical Repository," and of the "New England Medical and Surgical Journal." Reference must be made, in this connection, to the second volume (1849) of the Transactions of the American Medical Association, containing a Report on Indigenous Medical Botany by Dr. N. S. Davis, and more especially the carefully elaborated Reports on the Indigenous Medicinal Plants of South Carolina by Dr. F. P. Porcher, and on the Indigenous Medical Botany of Massachusetts by Dr. S. W. Williams, members of the committee of which Dr. Davis was chairman. In the seventh volume of the Transactions of the Association (1854) will be found a supplementary Report by Dr. Porcher, on the Medicinal and Toxicological Properties of the Cryptogamic Plants of the United States. At an earlier date (1821 and 1824) appeared a work in two volumes, by Stephen Elliott, LL.D., entitled "A Sketch of the Botany of South Carolina and Georgia." The systematic works on Materia Medica and Therapeutics, by American authors, constitute a full proportion of the Medical Literature of the country. The authors have been, with scarcely an exception, lecturers in medical schools, and the volumes issued by them are, in some cases, nearly the same as their prelections. Mention may here be made, in the order of their first appearance, of the treatises by, respectively, Drs. Chapman, Eberle, Dunglison, Frost, Harrison, Paine, J. R. Beck, Thos. D. Mitchell, and Wood. The Dispensatory of the United States of America, by Drs. Wood and Bache, as a comprehensive work on Materia Medica and Pharmacy, addresses itself to physicians, druggists, and apothecaries. Dr. Beck, in addition to his volume of posthumously published lectures, edited by Dr. Gilman, is the author of a small volume of an earlier date, entitled "Essays on Infant Therapeutics." Dr. John Bell, author of the present Report, has issued a "Dictionary of Materia Medica and Therapeutics," based on Brunde's Dictionary of Materia Medica and Pharmacy. The therapeutical additions and changes are numerous and radical, and include a notice of the medicinal value of the chief indigenous plants of the United States. Dr. John B. Biddle has sent out a small volume, bearing the title of a "Review of Materia Medica," designed for the use of students. Under the double head of Materia Medica and Hygiene, mention may, properly, be made here of three several works by Dr. Bell, viz.:—1. Baths and Mineral Waters; 2. Baths and the Watery Regimen; 3. Mineral Springs of the United States and Canada. On particular mineral springs, or on those of certain regions, we have the writings of Drs. Steele, North and Allen, on the Springs of Saratoga, and of Drs. Moorman and Burke on those of Virginia.

Dr. Beck, in his work on Infantile Therapeutics, to which reference has been made above, contests the claim advanced by English writers in favor of Dr. Robert Hamilton, of Lynn Regis, as the first to make free use of mercury in inflammatory complaints (1783.) This honor is, he thinks, mainly due to American physicians. It was in attempting to arrest the ravages of a dreadful epidemic (putrid sore throat) which prevailed in different parts of New England in 1735 and '36, that mercury was first used by Dr. Wm. Douglass, an eccentric physician of Boston. The preparation which he used was calomel. "This distemper did not well bear any other evacuations but mercurials," says Dr. D. The mercurial practice was systematized, and carried to the greatest extent by Dr. Jacob Ogden, a respectable physician of Long Island. About the middle of the last century, it was in common use in pleurisy, pneumonia, inflammatory rheumatism, and other phlegmasiæ. "The common practice," writes Dr. Beck, "was to give it in large doses when used as a cathartic, and when given as an alterant, or to affect the system, in doses of one or two grains; and this was combined with camphor, antimony, or opium, according to circumstances—a practice precisely similar to that of the present day." Dr. Thomas Bond, of Philadelphia, was the first to introduce mercury into general use in the practice of medicine. "He called it, emphatically, a revolutionary remedy, and presented it in all cases which resisted the common modes of practice. He gave it liberally in the cynanche trachealis. He sometimes cured madness, by giving it in such quantities as to excite salivation." Dr. Bond's professional life extended from 1734 to 1773.

The most valuable contributions to the Materia Medica, on the part of American physicians, are the Ergot of Rye, and Ether inhaled as an anæsthetic. For the introduction of the first into regular practice, with the view of aiding the parturient efforts of the female, we are indebted to Dr. John Stearns, residing at the time (1807) in Saratoga, New York. For the practical application of Ether for the relief of pain and suspension of sensibility by its inhalation, the world must acknowledge its obligations to Dr. Morton, a dentist of Boston, who was aided in his investigations and early trials by the advice and countenance of Dr. C. S. Jackson, of the same city. Other anæsthetics have been discovered since then and used in Europe, but no one of them imperils the life of the patient in so small a degree as ether. On the other hand, scarcely a month elapses without our hearing of a death caused by the inhalation of that favorite anæsthetic—chloroform—to produce the desired insensibility before an operation.

So soon as a few observations of facts or phenomena of a kindred character are made, the mind feels the necessity of classifying or arranging them methodically, under one head, so that, by a kind of mnemonics, the general title will suggest the separate particulars, and thus the memory is saved from the continual strain of calling up each individual fact, and its concomitant attributes or circumstance. Certain resemblances in physiognomy or in properties, are made the groundwork of the classifications in natural history; as in the cases of certain botanical and zoological classifications. Similar attempts, on a like foundation, have been made, to classify the functions of the several parts of the living organism in health, and the numerous aberrations of the functions from this standard in disease. So, also, we have classifications of the articles of the Materia Medica, which, for the most part, are framed on the basis of their direct sensible and physiological operations on the several organs of the economy. This mode of proceeding would be very convenient, and undoubtedly useful, if such an article produced a unit effect, or acted on one organ or on one organic apparatus only; but, so various are its immediate effects, that we soon find our classification to belong to Therapeutics, rather than to Materia Medica, and that under one division of the former we have several articles of the latter. and often, one of these articles obtains a place under different therapeutical divisions. And again, there are several articles of the Materia Medica which are of great power and efficacy, but the *modus operandi* of which on the organism is of such an occult kind, and so little, if at all, manifested by either evacuating, or exciting, or depressing effects, that we are unable to enlist them under any of the therapeutical divisions in the ordinary classifications, unless, in some cases, it be under the very general term of *alteratives*, which is itself an acknowledgement of a difficulty evaded but not overcome. On the present occasion, we, in common with all who teach or write on Materia Medica, feel this embarrassment; and if we attempt a classfication at all, it can have no claim to be considered as a scientific one, but only as at best an aid to memory.

We begin our report on the American contributions to Materia Medica and Therapeutics for the year 1857, with certain articles belonging to the class of

### SEDATIVES.

Of these, the article which most engaged attention during the year, and the medicinal effects of which make the nearest approach to novelty, is the indigenous plant known as

*Veratrum Viride—American Hellebore.*—The peculiar properties of this plant were known to the Aborigines; but the first regular account of its therapeutical value and application, was furnished by Dr. Charles Osgood, of Providence, in a paper contained in the *American Journal of the Medical Sciences*, vol. xvi. He pointed out, among other effects, the remarkable diminution of the heart's action, and reduction of the pulsation at the wrist to thirty-five beats in a minute. Dr. Tully, of New Haven, also experimented with the veratrum, and recommended it as a substitute for colchicum. It was thought, at this time, to be best adapted to gouty, rheumatic, and neuralgic disorders. So soon as we begin to speak of the immediate effects following the administration of this substance, we find an illustration of the remark made above, on the difficulty of classification in Materia Medica. The Veratrum Viride is used in substance, tincture, and extract. Its first effect, if a dose of four to six grains of the powder, or one to two fluidrachms of the tincture be taken, is to cause vomiting. At the present time, renewed prominence has been obtained for the veratrum, by the iterated efforts of Dr. W. C. Norwood, of South Carolina, whose zeal would have been more generally appreciated, but for the imputation of self-advantage or profit, to which he exposed himself by intimating, if not directly claiming, a superiority for his preparation of the tincture over that of others. A severe and apparently a too well deserved rebuke for this and another and more open specimen of quackery on the part of Dr. Norwood, is administered by Dr. Taliaferro, in the *Atlanta Medical and Surgical Journal.*

The veratrum viride, although most probably possessing certain constituents in common with the *veratrum album*, differs from this latter in one essential particular, viz.: in its exerting little or no action on the bowels, as an aperient. Dr. Horace Green (*American Medical Monthly*, Feb. 1, 1857,) under the head of "Selections from Favorite Prescriptions of Living American Practitioners," assures us, when speaking of the veratrum viride, that, if employed in the early stage, before the occurrence of organic lesions, it is peculiarly adapted to the treatment of pneumonia, pleurisy, rheumatism, typhoid fever, and pulmonary inflammation; and in the phlegmasiæ generally it is regarded as more or less valuable. It has never failed in his hands, when properly administered, to diminish arterial action. In one case of tuberculosis, a pulse of 120 was reduced to 58 beats in a minute, and kept steadily at that point day after day. Dr. Green adverts to the favorable experience of Dr. Carnochan in the use of this remedy, in the Emigrants' Hospital, New York. In one case, a pulse of 130 was reduced to 68 beats in fifteen hours; and in another of articular rheumatism, by the exhibition of the tincture, in doses of from five to eight drops every three hours, a pulse of 120 was reduced to 30 in fifteen hours.

The saturated tincture—the form in which the remedy is directed by Dr. Norwood—is "made by macerating eight ounces of the dried root in sixteen ounces of alcohol for at least two weeks."

With a view of procuring more entire sedative and antiphlogistic effects, Dr. Norwood advises a combination of the tincture of veratrum and syrup of squills in equal parts. He directs of this mixture, from four to six drops for an adult male, which is equal to two or three drops of the tincture, and the same of the syrup. There must be some mistake here. How is sedative action to be increased by diminishing the dose of the veratrum? and what compensation is furnished by the insignificant addition of two or three drops of the syrup of squills?

Nausea and vomiting, to an undue extreme or persistence, after the use of the veratrum viride, will be attended with prostration and great coldness of the surface. These symptoms are readily dispelled by the administration of an opiate, and of brandy or other alcoholic stimulants.

Dr. R. H. Jameson (*Trans. State Med. Soc. of Indiana*, 1857,) presents a clear and well-written statement of his experience respecting the therapeutical value of veratrum viride. He has used this agent frequently within the last three years; and he regards it as one of great power and certainty, and as applicable to many of the forms of disease which are constantly occurring. With Dr. Wood, he very justly, as we think, looks on veratrum viride as "a nervous sedative, which acts directly on that part of the nervous system governing the circulatory function, and not indirectly through a nauseant impression, as supposed by some observers." Both he and Dr. Norwood have noticed a marked diminution in the frequency of the beats of the pulse without the least nausea. Dr. Jameson declares this medicine to be an agent of great power and utility in pneumonia. He has seen its administration followed by a relief of all its distressing symptoms, in a degree equal to that which is known to follow blood-letting. It "increases expectoration, and indirectly restores the depurating functions of the liver and kidneys. In different cases it may be given with benefit either after blood-letting, or along with quinine. A good mode of prescribing it for adults is the following:—

℞.—Tinct. Verat. Vir., (Norwood's,) f℥j.

Nitrat. Potass. Pur. - - - - ʒj.

Aquæ - - - - - - - - - ℥j.

M. Dose.—A teaspoonful every few hours, to be alternated with small doses of mercury, if deemed advisable." If the remedy fails to produce the desired relief, the dose may be gradually increased until it is doubled; but this is seldom necessary. Dr. Jameson cautions against our attempting to bring down the pulse suddenly by large doses of veratrum; and at all times we ought, he thinks, to deprecate its reduction below the normal standard.

"One considerable advantage which this remedy possesses over tartar emetic, is that it does not purge; which is often a serious objection to the use of antimonials in pneumonia." Dr. Jameson need not be told that this effect is prevented by the addition of a little opium, which is both admissible and beneficial in the phlegmasiæ generally, and which has the further advantage of preventing nausea and vomiting—results, by-the-way, which are common enough after the first or even second doses of tartar emetic; the medicine is then tolerated without nausea or a feeling of depression so long as the inflammation persists.

Veratrum viride is regarded by Dr. Jameson as "a good remedy in many cases of typhoid fever, especially where there is a dry, hot skin, parched, dark-brown tongue, and a quick, sharp pulse, with sleeplessness. Some of you will doubtless recollect that Dr. Graves recommends tartar emetic in such cases, to procure rest. Six drops may be given every four hours with nit. pot.; and if there is irritability of the stomach or bowels, a little morphine may be added. If the pulse is not reduced in twenty-four hours, the dose may be increased." Dr. Jameson does not deem it advisable to reduce the pulse of the adult lower than 70 beats in the minute.

Dr. Jameson has employed veratrum viride in two cases of puerperal fever, with what he considers favorable results. "With the verat. virid. the pulse can be as much reduced in frequency and kept down as long as the practitioner desires. With it puerperal fever may be, if not arrested at its outset, modified in its course, and effusion, that most fatal of its consequences, prevented."

Dr. Jameson regards veratrum viride as a safe medicine when administered with proper precaution. He is not inclined to attach any weight to the formidable accusations brought against it by Dr. W. A. Brown, of the tendency of its operation to procure abortion.

Our knowledge of Dr. Jameson's paper is derived from the August number (1857) of the *North Western Med. and Surg. Journ.*

Dr. H. Levezey, of Pennsylvania, (*Bost. Med. and Surg. Journ.*, March, 1857,) expresses himself, in strong terms, in favor of the veratrum. His experience enables him "to speak of it as one of the most certain sedative diaphoretics belonging to the Materia Medica, as an unsurpassed expectorant in all diseases of the respiratory organs attended with much febrile or inflammatory action, where this class of agents is indicated; and as an invaluable *arterial sedative*, for which property it stands unequaled and unparalleled among all the therapeutic agents." The preparation of the plant used by Dr. Levezey is the fluid extract prepared by Tilden & Co., in the dose of five drops, increased till an effect is produced, and it is then to be diminished.

Dr. Levezey goes on to speak of the sedative and antiphlogistic powers of the medicine, as prompt in inflammatory croup, pneumonia, or pleurisy, after the administration of calomel with ipecac. or jalap, in equal quantities, repeated every half hour or hour, until catharsis, with or without emesis, is produced. The effect of the veratrum under these circumstances would be, we might suggest to Dr. Levezey, quite auxiliary. He adds, however, that "the arterial action is more *permanently* reduced by this agent than by the lancet; the farther progress of the inflammatory process is subdued, expectoration is promoted, and a cure results often without the aid of other medicines."

Dr. G. W. Boerstler, of Lancaster, Ohio, (*West. Lancet*, May, 1857,) after some preliminary observations of a doctrinal nature, proceeds to give, in the following terms, his experience of the morbid conditions in which he found veratrum viride to be serviceable:—

"In all cases wherein it was desirable to control the perturbed state of the heart, the veratrum has never disappointed me, without one single unfavorable symptom resulting from its use. In no instance, however, have I brought down the heart's action to a normal state, without my patient complaining of some degree of nausea. After the heart's action was curbed, and the medicine continued in similar doses, no sickness of stomach resulted. I have given it to patients for two months, and never witnessed any cathartic action, nor did I observe any influence on the glandular system. As a curb to the inordinate action of the heart, it is truly *invaluable.*"

Dr. Boerstler terminates his short paper by a remark which is both just in itself, and apposite to the present subject. "Believing as I do," he says, "that the *vis a tergo* of the heart, is neither the first link, nor yet the essential phenomenon in inflammatory diseases, I hold the veratrum, not as a curative agent, but as a most desirable adjuvant."

It is an undoubted fact in pathology, that the heart, in its excited action, in inflammation and fevers, is simply responsive to some local irritation or blood poisoning; and, as such, furnishes a symptom—a very important one, it is true, but only a symptom—of a pre-existing morbid process. We may, by contra-stimulants or sedatives, allay inordinate cardiac action; but we, in so doing, only abate or suspend an effect, and do not remove a cause. The sanguine advocates of digitalis, at the beginning of the present century, gave into the same mistaken pathology as the eulogists of veratrum viride do at the present time. Then, the paramount object was to lessen the frequency of the pulse, and, as an alleged effect of this, to abate or remove the hectic fever; and this result could be obtained, it was thought, by digitalis, which was found to produce such a marked sedative influence over the circulation. The idea did not present itself, that the cardiac irritation and the hectic fever are maintained by the presence of pulmonary tubercle, over which digitalis has as little control as so much water. Dr. Green, in his praise of the veratrum viride, already quoted, speaks of its reducing the pulse in a case of tuberculosis, and of the reduction, (from 120 to 58 beats in a minute,) being kept at that point day after day; but he does not, for he could not, in fact, tell of any benign influence having been exerted on tuberculosis, and still less on the formed tubercle itself.

Common inflammation, it is true, is more affected by the state of the circulation than such heterologous formations as tubercles; but this inflammation is far from being merely a hydraulic problem, to be solved by ascertaining the propelling power of the heart, and the size and resistance of the vessels acting on the circulating blood. In the reticulated capillary tissue, in which inflammation takes place, a new structure, almost a new organ, is formed. The elements for pathological study are now to be sought in the blood-vessels of the part, in the tissue, or parenchyma intermediate between these vessels, in the altered movements of the blood, and in the altered qualities of this fluid. In addition, we meet with other changes, viz.: in innervation or nervous function, and in the general circulation, and especially in the action of the heart. It must, however, be remembered, that these last are concomitant, if not consecutive, rather than essential parts of the phenomena of inflammation, which, as far as it consists in the destruction of old parts and the formation of new products, takes place in vegetables in which there is neither innervation nor circulation.

We are not yet in possession of the requisite experiments or of positive observations, to allow of our ascertaining on which of the two classes of nerves—the sensitive proper, or the nutritive, the veratrum viride exerts its chief power. Nor have we any, or scarcely any, carefully recorded clinics to show its therapeutical effects, either in shortening the entire period of inflammation, or in modifying the successive stages of this process. It is with the hope of aiding future inquirers, by fixing their attention on the real problem to be solved, that we have ventured to make the preceding remarks on the pathological conditions, which are said to be changed by such remedies as the one now under notice. As yet, we are quite unable to participate in the sanguine belief of those who, like Dr. Taliaferro, assert for the veratrum viride a superiority over the lancet in the treatment of inflammatory diseases. This writer (*Atlanta Medical and Surgical Journal*, March, 1857,) tells us: "The great advantage this remedy possesses over the abstraction of blood is, that its effects are far more decided, and not followed by the reaction which almost invariably succeeds venesection. Not only this, but the effects of the remedy may be continued for days and even weeks, successively, without unpleasant consequences; whereas a frequent repetition of blood-letting would, in most cases, be injurious." Dr. Taliaferro alleges the superiority of veratrum over antimony, as an antiphlogistic, because it is wholly free from drastic properties. Much stress cannot be laid on this opinion, until it is positively confirmed by a series of comparative trials. This qualifying remark of ours will apply, also, to another assertion of Dr. Taliaferro; that, "in the treatment of pneumonia it (the veratrum viride) is unquestionably the most reliable agent we have. When the tissue of the lung becomes inflamed, febrile excitement running high, the breathing short and quick, the proper use of veratrum soon quiets the excited action of the heart, and the blood is sent smoothly and gently along its channels." It must be borne in mind, however, that more formidable obstructions are to be overcome in the case of an inflamed lung than those interposed by an excited heart and hurried flow of blood in the vessels of the phlogosed part. The restoration of a hepatized lung to its normal condition and duty, is not resolvable into a question of *plus* or *minus* of cardiac and vascular action. But, in justice to Dr. Taliaferro, we must add that, while he claims for his favorite medicine, if freely used at the beginning of pneumonitis, the power of cutting short at once its progress, and while he places his principal reliance upon it in the treatment of this disease, he believes, still, "that important adjuvants are found in the use of opium, aconite, and calomel." He omits tartar emetic, the most reliable, in our belief, of all the drugs administered in pneumonia.

Dr. Taliaferro gives the outlines of a case of inflammation of a portion of the lower lobe of the right lung. The pulse, which was upwards of 100, full and bounding, was reduced, in the lapse of six hours, under the influence of the veratrum, to 70; the skin, from being burning hot, became "pleasant and moist." The first dose of the medicine was ten drops, it must be supposed of the tincture, to be followed, in three hours, by six, and in three hours more, by five drops, if the patient was not nauseated. After this period had elapsed, and the above good effects had been obtained, the Doctor gave his patient "six grains of calomel, combined with ten grains of *pulv. Doveri*, that he might rest well through the night." Not relying on this additional prescription, the veratrum was ordered through the night in four-drop doses. "The ensuing morning," continues Dr. Taliaferro, "we found him with no fever; pulse beating naturally; and expectoration free and easy; free from pain, except when coughing; auscultation revealed an evident subsidence in the inflammatory action of the lung. It is unnecessary to trace further the change from day to day; suffice it to say, that the veratrum was continued with no other medicine, save a Dover's powder at night, with a rapid subsidence of all diseased action; so much so, that upon the fourth day of his attack, he was able to walk upon the street; his medicine, in the mean time, being continued. The patient was never the least nauseated." The reader is left to draw his own conclusions as to the part performed by the veratrum in the curative means employed in this case.

Dr. Taliaferro, looking only to the element of accelerated circulation in typhoid fever, finds, in veratrum, a remedy not only serviceable in the commencement of this disease, but, also, in its latter stages, "reducing frequently the feeble, fluttering pulsation of the heart from 100 to 70 or 80, thereby changing an abnormal to a normal circulation." In a subsequent communication, in the *Atlanta Journal*, Dr. Taliaferro advises much care and circumspection in administering veratrum viride in hepatization of the lungs. In this state there is a much smaller surface than usual for the inspired air to produce the desired oxygenation of the blood—a process which would be still further impeded by an undue retardation of the circulating fluid in the lungs.

Dr. U. G. Mitchell Walker, of Alabama, in "A Few Words in Defence of Veratrum Viride," (*South. Med. and Surg. Journ.*,) expresses his belief that this medicine is "a great adjuvant when we wish to keep up the sedative influence produced by the lancet." It is, he thinks, particularly adapted to pneumonia, such as the disease now exhibits itself in that region of country, viz.: in its being of a type intermediate between the sthenic and asthenic, and not requiring the treatment generally deemed necessary in true sthenic pneumonia. Patients under the use of the veratrum viride are represented to "recover very rapidly, and without any of the unpleasant symptoms often noticed after active cathartics and blood-letting have been employed." So, also, by its sedative character it allays the nervous excitement in this disease, which often calls for opium. Dr. Walker advises the remedy (tincture of veratrum viride) to be administered, at first, in doses of four or five drops; or, when we have reason to believe that the patient will be easily affected, in as small a quantity as two or three drops, with a little sweetened water; the dose to be repeated every hour, with an increase of a drop at each dose, until the patient is brought under the influence of the medicine.

We go into the year preceding that of our retrospect to notice a paper by Dr. Hutchinson, of Indiana, read before the Hendricks Co. Med. Society, Nov. 1856, and published in the *Western Lancet*, February, 1857. He gives an account of five cases of pneumonia, in which the veratrum was used with success: in some after other remedies had been used; in some it was the only article.

This gentleman, like Dr. Tully, believes that "the effects of veratrum on the circulation are, to a great degree, like colchicum; the effects on the bowels are very different. In no instance have I found veratrum to purge. Like colchicum and digitalis, its powers are cumulative, frequently requiring several doses to bring down the pulse, or to produce emesis. Its influence in cleansing the liver is greater than that of any other article with which I am acquainted. It is best adapted to cases accompanied with a frequent pulse, and those in which a good deal of nervous irritation exists. The pulse produced by veratrum is *sui generis*, and when once recognized is easily recognized again. For convenience I have styled it the *veratrum* pulse. I have frequently brought down a pulse of 130 per minute as low as 60, and sometimes as low as 40. It has become fashionable to administer it in conjunction with paregoric and spirits of nitre. I do not know that the combination is of any value in aiding the effects of the remedy. The dose is easier proportioned by giving it single. It is highly spoken of in rheumatism. There is a case of cynanche trachealis cured by it, reported in the September number of the *Medical Observer*. I have no doubt of its value in this disease, from my experience with it in croup, and I am in the habit of adding it to my expectorant mixtures in bronchial irritation."

We give insertion here to a case of croup treated by Dr. Hutchinson, and related by him in the *Western Lancet*. His closing observation merits particular notice, as showing that the sedative effects of the veratrum viride are not owing to the nausea it often produces. The first stage of sedation, both in the case of this drug and of tartrate of antimony, precedes the nausea, which is, in fact, an effect,—a symptom, and not a cause.

"March 23, 1855, 8 o'clock A.M.—Was called to A. Greeson, aged three years; patient had been hoarse for the last two days. Breathing whistling; voice stridulous; pulse very frequent, at least 150 per minute, and quick. Tonsils covered with exudations of lymph. Gave alum emetic, according to the practice of Dr. Meigs; left four doses more, to be given every twenty minutes, till emesis took place. Called again in two hours; he had taken all the alum without vomiting; pulse still frequent; breathing whistling; stupor approaching. Gave tinct. veratrum viride, five drops, every twenty minutes. Returned to see him again in two hours; had taken four doses of veratrum; slight vomiting was produced; pulse not so frequent; breathing a little easier; still inclines to stupor. Abstracted four ounces of blood from a vein at the ankle. Continued the veratrum every half hour. Called again in two more hours; pulse down to 60 per minute; swelling of tonsils subsiding; breathes easy; does not incline to stupor; takes notice of objects around him; has not passed any urine for nearly twenty-four hours. Gave calomel and squills, of each one grain; continue veratrum, five drops every hour.

"Called again at 12 o'clock midnight. Has vomited a little; pulse 80, soft; breathes easy. Gave calomel to move the bowels, and continue veratrum.

"24th—8 o'clock A.M.—Nearly free of croup symptoms; continue veratrum in smaller doses. Give cathartic and diuretic, as he has passed but a few drops of urine. He required no further treatment, as he rapidly recovered, and is yet living.

"The most remarkable circumstance in this case was the quantity of veratrum administered, with the happiest result. He took, in twenty-four hours, one ounce of the tincture, without producing vomiting to any extent; but it speedily reduced the inflammation of the throat and air-passages. I have since used it in other cases of croup, with the best effects."

Dr. C. H. Murphy (*St. Louis Med. and Surg. Reporter*, March, 1857,) states that he has used veratrum viride in twenty-one cases of pneumonia, without failing in any instance to produce its sedative effects, and with the loss of but one case, and that he attributes to "exposure to cold forty-eight hours after the pulse had been reduced to 60 per minute." Dr. Murphy relates the outlines of three cases of this disease, in the first of which he began the treatment, on the second day, by directing "calomel and ipecac., each one grain, every three hours until it acts, together with tinct. veratrum viride, gtt. vii., every three hours, alternately. The dose of the latter to be increased one drop in the dose until nausea is induced." Calomel and opium, in doses of one half the strength just indicated, and the tincture were continued on the third day. On the fourth day (of the disease) the veratrum was continued, as it was on the two following days. On the seventh day of the disease, and the sixth of treatment, convalescence was established, and the veratrum was discontinued. In its place tincture of the *Sanguinaria Canadensis* was given three times a day. The second case of pneumonia, consisting of inflammation of the inferior lobe of the right lung, had lasted five days, and had been treated with calomel and ipecac., and blisters, before it came under Dr. Murphy's charge. He directed calomel and ipecac., of each one grain, every three hours until the bowels were moved; and, alternately with this, tincture of veratrum viride every three hours, increasing one drop each dose until it nauseated. On the following day, the dose of veratrum was diminished, and in the afternoon it was increased. "The patient sleeps but very little." On the next day there was great relief; the sleep had been good. The medicine was continued. The same report of amelioration and treatment on the succeeding day. Convalescence began on the seventh day of treatment by Dr. Murphy, and the twelfth day of the disease. In the third case, after the use of calomel fifteen grains, and jalap three, divided into three powders, one to be taken every two hours, the veratrum was the only therapeutical agent employed, except a blister to the side. The dose of the tincture was eight drops every three hours, increasing one drop each dose till it nauseated. The medicine was increased by the next day, to sixteen drops at a dose, and, in the following one, reduced to eight drops, at which it was continued. On the seventh day of treatment, the patient was pronounced convalescent.

In the number of the journal just quoted from, we find an account of the treatment of seven cases of acute inflammation, by Dr. Watson, originally inserted in the *Nashville Journal*. In all of these, of which five were of pneumonia, the veratrum was given with satisfactory results. The first patient, a negro girl, "aged 17 years, has been successively bled, cupped, cauterized, blistered, etc., with but slight benefit. Believing that she would soon die, unless the arterial excitement could be subdued," Dr. Watson "gave 12 drops of the tincture of veratrum. In ten or fifteen minutes there was a profuse secretion of saliva, and in half an hour, vomiting, and reduction of the pulse from 130 to 60 per minute. The retching and sickness lasted two hours, after which there ensued a copious bronchial secretion. On the following day, the pulse had regained its former frequency. The tincture of veratrum was ordered to be repeated every two hours; but the second dose having produced the desired effect, the medicine was discontinued. From this time the patient rapidly recovered; it being only necessary to repeat the medicine on one other occasion."

W. Thomas, aged 25 years, had suffered from an attack of acute rheumatism; "had used the lancet, colchicum, opium, lemonade, etc., etc., with but little benefit; gave the veratrum with the highest success. The medicine was continued for three successive days, after which time he was relieved."

In the case of a child, aged 18 months, who was seized with pneumonia, tartar emetic, the warm bath, calomel, etc., had been used, but with what results is not stated. Dr. Watson "gave one drop of veratrum; waited two hours; gave three drops. Patient became calm, and slept. The pulse was reduced from 140 to 40 per minute." The Doctor was alarmed, but in due time "the pulse rallied, and in eight or ten hours the effects had entirely passed off; the remedy was repeated in two-drop doses on two other occasions. The child recovered rapidly." In another case, in which the pneumonia had lasted two days before treatment was begun, Dr. Watson prescribed the tincture of veratrum in two-drop doses, every two hours, "increasing [to?] three drops upon each repetition." After the third dose, free and repeated vomiting was produced, with the ejection of large quantities of mucus. The sickness of stomach having subsided, a copious diaphoresis came on, accompanied with free expectoration, and the patient rapidly recovered without any further treatment. The age of the patient was 26 years.

Case third was relieved by the same means, with equal promptitude, after a second dose. In one of the cases of pneumonia, the pulse proved intractable under the use of the lancet, nor could nausea be caused by tartar emetic. Equally unavailing was the tincture of veratrum, in doses of 16 drops repeated every two hours, in three successive days, without the slightest effect. The patient's recovery, as Dr. Watson thinks, was due to a copious bleeding resorted to on the seventh day. He does not seem to be aware of the true antiphlogistic or contrastimulant effect of tartar emetic, in his looking for its nauseating effects as a means of abating inflammation. Need we say that this medicine acts most beneficially in cases of phlogosis, without its giving rise either to nausea and vomiting, or to purging, or to an increase of any secretion? Its being tolerated in this way, is both an evidence of the safety of continuing its use in increased quantities, and of the persistence of the inflammation. So soon as this morbid state is removed or greatly diminished, the toleration of the stomach for the tartar emetic ceases, and nausea, vomiting, and sometimes catharsis supervene.

Dr. D. L. M'Gugin, of Keokuk, Iowa, (*Iowa Medical Journal*, No. 1, 1857,) adduces his experience of the efficacy of veratrum viride in four cases of pneumonia in measles, and in two cases of acute rheumatism. In the first case of pneumonia of the lobular variety, in a little girl of a nervous temperament, calomel and tartar emetic had been used for several days without lessening the action of the heart. The tincture of the veratrum was then ventured on, at first in two-drop doses in syrup of squills, and afterwards in four drops every three hours,—with the effect of sinking the pulse from 140 to 100 beats in the minute, and of increasing the expectoration, moderating the cough, and bringing out moisture of the skin. "The tincture was continued a few days, during which the pulse was controlled by it, and the patient rapidly convalesced.

The next case was that of a stout Irish girl, aged 14 years, laboring under double pneumonia. The pulse was 130. Four drops of the veratrum tincture were given at first, and six drops afterwards. In twelve hours the pulse was reduced to 86. Great relief of all the disturbed functions was produced. A suspension of the use of the tincture during the night, was followed, toward morning, by renewed frequency and quickness of the pulse; but on fresh recourse to the medicine, the desired reduction was obtained. After an attack of six days' duration, the patient began rapidly to convalesce." The third case was that of a young man who had suffered from a previous attack of pneumonia of the right side. He was afflicted this time to such a degree, "that his face was not only livid, but rather cyanotic." Pulse, 130; respiration hurried and laborious; dull sound on percussion of the right side.

The treatment consisted in the administration of calomel combined with tartrate of antimony, in small and repeated doses,—quantity and intervals not stated,—and the application of stimulating embrocations over the right side of the chest, corresponding with what Dr. M'Gugin supposed to be inflamed pulmonary pleura of the lower lobe. The bowels were moved by a mild cathartic. After this the patient was ordered tincture of veratrum viride in doses of four drops, with the same quantity of simple syrup of squills, every two hours, to be increased until nausea and vomiting should take place. In twelve hours the pulse came down to 80, and was full and soft; the skin covered with perspiration; expectoration easy and abundant; respiration twenty; the face pale; the pain on the right side in the region of the lower lobe, was relieved. Suspension of the veratrum was followed by a renewal of the inflammatory symptoms. On its second resumption, its use was continued until the patient was decidedly convalescent.

The fourth case was the more interesting, as it showed that, even in a broken-down constitution, pneumonia may be successfully controlled by veratrum viride. The dose (of the tincture) was at first four drops every four hours; and, after a period of twenty-four hours had elapsed, without the desired reduction of pulse, five drops, at intervals of three hours, were directed. Nausea and an effort to vomit followed the exhibition of the second dose, and the pulse fell to 80. "There was copious perspiration, with warm skin; the breathing, sixteen; the expectoration free, and, for the first time, rusty; the bowels were freely moved; the urine copious, and nearer the normal color, and there was an entire freedom from pain. Convalescence was ere long established; the tincture having been continued in less doses, and at longer intervals."

Dr. W. R. Handy, of Baltimore, (*Boston Med. and Surg. Journ.*, April, 1857,) gives the details of a very interesting case of pneumonia, in which, although it ended in death, the remedial effects of the veratrum viride were very conspicuous. Dr. Handy and Dr. Whitridge, who attended with him, had recourse at first to a vigorous antiphlogistic course, but without effect. It consisted of venesection, leeching, blistering, calomel, nitre, and antimony. This treatment was pursued during two weeks. "And yet," writes Dr. Handy, "with the whole we failed to act upon the skin, either in diminishing its heat and dryness, or producing the least noticeable degree of moisture. We failed to reduce the frequency of the pulse, though its hardness succumbed. We failed to destroy, or even alter the tenacity or viscidity of the expectoration." The patient was a colored woman, aged 30. Recourse was had to the veratrum, and the first dose of the tincture (Tilden & Co.'s preparation) was five drops, which was repeated three times, at intervals of about four hours. The first dose was three drops, "so that from 2 o'clock of the one day, March 24th, to 11 o'clock of the next day, (twenty-one hours,) five doses of the veratrum had been given, and the pulse fell from 120 to 94, (reduction, 26 strokes,) and the respiration was entirely free." In five minutes after the first dose, the patient was in a profuse perspiration. When Dr. Handy saw her, about three hours afterwards, and before the dose had been repeated, he "found her literally in a harvest sweat, and the sputa were more free and less viscid. The first dose did not reduce the frequency of the pulse. "In the twenty-one hours (March 25th) the veratrum was given in three-drop doses, and but five were taken. The perspiration was kept up; the pulse had fallen to 80 strokes; the expectoration all the time free and abundant; and the respiration also still free." A diminution of the dose to two drops, at intervals, in twenty-one hours, (March 26th,) was followed by a rise of the pulse to 112; but on the increase of the dose to three and to five drops, it fell to 88; and on the 28th of March to 64, accompanied with great prostration. The patient soon rallied from this under the use of laudanum and compound spirits of lavender internally, and warm applications externally; and the pulse rose at 4 P.M. to 80. On the first of April, the three-drop doses seemed to have lost their effect, and four drops were given. After the first dose of the latter, the patient became sick, and a free perspiration broke out, when the three-drop doses were resumed. Taking into account the copious and constant expectoration and perspiration, which acted as a drain and weakened the patient, and increased the tendency to a frequent pulse, Dr. Handy and Dr. Whitridge agreed to stop the use of the veratrum, and to use in its stead tincture of digitalis in ten-drop doses. One of these was given on the second of April, and three on the following day; and hydrocyanic acid, in one-drop doses, was also given, at intervals, for the cough. The pulse, so far from being reduced under this treatment, was increased four strokes in the day. The patient was evidently failing on the fourth. The digitalis was withheld on the fifth. The case terminated fatally on the following Tuesday; but on what day of the month we are not told.

Dr. Handy, in his "remarks" at the close of his narrative of the case, thinks that it shows veratrum to be a prompt and most powerful diaphoretic, and almost equally so a prompt and efficient expectorant, and a decided and reliable arterial sedative. It is a noteworthy circumstance that the pulse was reduced 24 strokes in the minute before nausea was produced. "This case enables us also to fix the dose (as far as evidence in a single case can be considered trustworthy) which may be administered with safety." The contrast between the veratrum and the digitalis is decidedly, Dr. Handy thinks, in favor of the former, although he admits that the objection may be plausibly urged that the digitalis was not good. Let us add, that a fair trial of the sedative properties of this last-mentioned article was not made by administering the tincture in such a small dose as ten drops.

In the first case of rheumatism mentioned by Dr. M'Gugin, (*Iowa Med. Journ.*,

No. 2, 1857,) that of a lady, colchicum, nitre, opium, and calomel had been used. The exhibition of the veratrum was followed by a diminution of pain, and of all the other distressing symptoms. "The case came forward to speedy convalescence." The next was that of a resident of Virginia, of a plethoric habit, and who had been similarly affected several times. He was unable to turn in his bed, and the least attempt by others to move him was productive of great pain. "Calomel, opium, colchicum, and the usual remedies in such cases, were tried in a heroic manner, and yet the case threatened to outlive the allotted term of Dr. Watson, 'of six weeks' duration.'" After the veratrum had been taken, "the pulse soon gave evidence of its powers; the skin became relaxed, and the bowels poured out largely of highly offensive matter. He continued to improve, and says his disease is 'broken up.'" In this case the medicine would seem to have had an aperient effect.

Dr. M'Gugin, in giving his creed on the subject of the medicinal operation of the veratrum viride, is "convinced that, as an arterial sedative, we have no superior, and that it fills a void in the list of our therapeutic agents which we have felt; and from the want of such agent we have all along struggled." He thinks "there is much more certainty in its therapeutic action, than in a majority of the drugs now used. Its diaphoretic powers were very manifest, and its expectorant influences were observed by Prof. Allen, as well as myself, whose confidence in the *veratrum* is equally strong with my own."

Dr. Hill, of Illinois, (*North Western Med. and Surg. Journ.*, May, 1857,) relates the case of a Miss B——, with remittent fever, for which, on the first day of his visit, he directed a purgative of compound extract of colocynth and calomel, to be followed, if need be, in six hours, by sulphate of magnesia; he gave also the effervescing draught every hour, in combination with spirits of nitre and a little tartar emetic while the febrile stage lasted; and he left sulphate of quinia—doses not stated—"to be given at short intervals from the time that the intermission should occur." By mistake, the quinine was given in the course of the day without waiting for the desired intermission. One must certainly marvel at the strength of Miss B.'s stomach, which could receive without revolt, calomel, compound extract of colocynth, Epsom salts, effervescing draught, with spirits of nitre and tartar emetic, and, to crown all, sulphate of quinia—and this in one day! Dr. Hill, on calling the next morning, found that the cathartic had operated well, and also that "fever, wild and high, coursed through every vein" of his patient, with the additional features of active cerebral congestion and constant delirium. "I wanted," writes Dr. Hill, "an agent to reduce the force and volume of the circulation, and that promptly, too." He gave, with this view, "two doses, of five drops each, of the tincture of veratrum at an interval of half an hour between them, which produced emesis, followed by diminished force and frequency of the pulse; removal of the delirium and the supervention of drowsiness. Ordered three drops of the tincture, a little spirits of nitre every hour and a half till a complete intermission should occur. Next morning the patient was found sitting up in bed; pulse soft and moderately full, and about the healthy standard in frequency; skin cool, and slightly moist. A fine opportunity was now offered for the administration of quinine, by which the cure was complete."

Dr. Hill has given the veratrum in almost every case occurring in his practice, in which "a control over the force, frequency, and volume of the circulation was principally indicated," and to a considerable extent "its use has been followed by the expected effect." He has "seen it followed by alarming depression, requiring a prompt recourse to alcoholic or opiate stimulus, to prevent the apparent syncope to which the patient was hastening."

"*On the Use of Veratrum Viride in Puerperal Fever,*" is the heading of the "report of a case in the service of Prof. Barker, of Bellevue Hospital, New York, by Reuben Cobb, M.D., House Physician." We insert it entire from the pages of the *American Medical Monthly* for November, 1857, as the closest clinical record yet published of the symptoms and progress of the disease during the period in which the medicine was administered :—

"Kate Short, aged 23 years, fell in labor in full term at 2 o'clock P.M., February 25th, and was delivered of a healthy child at 8½ o'clock on the morning of the 26th. Nothing unusual occurred in her labor, except that the second stage was somewhat prolonged. Placenta came away in due time, and was not followed by hemorrhage. First pregnancy.

"February 28th, at 8 A.M., she was seized with a very severe chill, followed by increased frequency of pulse, and pain over hypogastric region, extending as high up as the umbilicus. This pain was very much increased by taking a full inspiration, or by the application of pressure. Tympanites very considerable. Discharge abundant and very offensive. Pulse 140. Respirations twenty-four.

"At 1 o'clock P.M., Dr. Barker saw her, and recommended that she should be transferred to the Fever Wards, and put on the use of the Tinctura Veratri Viridis.

"At 2 o'clock P.M., after having been removed to the Fever Wards, her pulse was 140. Respiration 24. Pain over hypogastric region intense. Tympanites very considerable. Discharge abundant and very offensive. No mammary secretion. Dr. Barker requested that she should be seen hourly by one of the House Staff, and that her condition, as to the state of the pulse, respiration, and other symptoms, and the dose of the veratrum viride given should be recorded at each visit. The following is the record thus kept:—

| | *Hour.* | *Pulse.* | *Resp.* | *Drops.* | |
|---|---|---|---|---|---|
| *February 28th.* | 2 P.M. | 140 | 24 | 10 | |
| | 3 | 127 | 22 | 10 | |
| | 5 | 140 | 22 | 10 | |
| | 6 | 132 | 12 | 10 | |
| | 7 | 120 | 20 | 10 | |
| | 8 | 80 | 20 | 9 | Bowels moved once. |
| | 9 | 75 | 16 | | Vomited a greenish colored fluid. Bowels loose. |
| | 10 | 66 | 16 | 4 | Vomiting ceased. Bowels moved once. |
| | 11 | 65 | 22 | 7 | |
| | 12 | 58 | 13 | 2 | |
| *March 1st.* | 1 A.M. | 64 | 52 | 6 | Respiration very irregular. Inclined to sleep. |
| | 2 | 58 | 25 | 2 | Sleeping. |
| | 3 | 59 | 21 | | Hiccough and headache. |
| | 4 | 60 | 18 | 1 | Hiccough still continues. |
| | 5 | 66 | 20 | | Severe headache. Vomited a greenish colored fluid. |
| | 6 | 66 | 21 | | Headache severe, and very restless. Vomited several times within last hour. Hiccough. |
| | 7 | 58 | 20 | | Vomited once since last visit. Vertigo and headache. |
| | 8 | 52 | 28 | | Sleeping. |
| | 9 | 60 | 19 | | |
| | 10 | 68 | 21 | 1 | Slight hiccough. |
| | 11 | 70 | 23 | 2 | |
| | 12 | 80 | 28 | 3 | Tenderness over abdomen, marked. Tympanites somewhat diminished. Discharge dark, bloody, and very offensive. |

| | *Hour.* | *Pulse.* | *Resp.* | *Drops.* | |
|---|---|---|---|---|---|
| *March 1st.* | 1 P.M. | 80 | 20 | 4 | Visit of Prof. Barker |
| | 2 | 92 | 24 | 8 | |
| | 3 | 76 | 24 | 8 | Face flushed. |
| | 4 | 76 | 28 | 9 | Sleeping. |
| | 5 | 68 | 28 | 8 | Sleeping. |
| | 6 | 66 | 28 | 8 | |
| | 7 | 68 | 26 | 6 | Slight hiccough. Bowels moved once. |
| | 8 | 66 | 18 | | Vomited a greenish colored fluid. |
| | 9 | 68 | 24 | | Vomited once since last visit. |
| | 10 | 60 | 28 | | Sleeping. |
| | 11 | 64 | 28 | | Still sleeping. |
| | 12 | 66 | 28 | 2 | Sleeping still. |
| *March 2d.* | 1 A.M. | 56 | 32 | | |
| | 2 | 70 | 24 | 3 | Complains of pain in left thigh. There is slight swelling, and along its internal surface, over the course of the veins and lymphatics, the tenderness is so great that she can scarcely bear the lightest touch. Tenderness over abdomen still continues. Slight tympanites. Discharge abundant, dark, bloody, and very offensive. No mammary secretion. |
| | 3 | 76 | 24 | 4 | |
| | 4 | 65 | 20 | 3 | Sleeping. |
| | 5 | 78 | 22 | 8 | |
| | 6 | 68 | 22 | 4 | |
| | 8 | 64 | 24 | 4 | |
| | 9 | 72 | 24 | 6 | |
| | 10 | 64 | 28 | 2 | Bowels moved once. |
| | 11 | 72 | 28 | 6 | |
| | 12 | 70 | 24 | 5 | |
| | 1 P.M. | 64 | 24 | 3 | |
| | 2 | 60 | 20 | | |
| | 3 | 64 | 24 | | |
| | 6 | 68 | 28 | 3 | |
| | 7 | 72 | 28 | 5 | |
| | 9 | 80 | 28 | 6 | Face flushed. |
| | 10 | 80 | 26 | 6 | |
| | 11 | 80 | 28 | 8 | |
| | 12 | 80 | 28 | 10 | Sleeping. |
| *March 3d.* | 1 A.M. | 80 | 29 | | Vaginal discharge now ceases to be offensive. No mammary secretion. Tympanites still remains. Tenderness over abdomen still continues, though not so well marked. Tenderness and swelling in left thigh still continue. |
| | 2 | 78 | 28 | 10 | |
| | 3 | 80 | 28 | 8 | Slight hiccough. |
| | 4 | 72 | 20 | 4 | |
| | 5 | 68 | 28 | | Vomited a greenish colored fluid. Headache. Hiccough. Bowels moved twice. |
| | 6 | 64 | 24 | | |
| | 8 | 60 | 24 | | |
| | 10 | 68 | 24 | 5 | |
| | 11 | 70 | 24 | 3 | |
| | 12 | 76 | 28 | 6 | |
| | 1 P.M. | 80 | 28 | 6 | |
| | 2 | 80 | 22 | 8 | |
| | 3 | 76 | 30 | 4 | |
| | 4 | 76 | 26 | 5 | |
| | 5 | 72 | 32 | 4 | Sleeping. |
| | 7 | 64 | 32 | 2 | |
| | 8 | 72 | 28 | 5 | |
| | 9 | 68 | 30 | 4 | |
| | 10 | 68 | 28 | 3 | |
| | 11 | 72 | 28 | 5 | |
| | 12 | 70 | 30 | 7 | Sleeping. |
| *March 4th.* | 1 A.M. | 72 | 32 | 8 | Tenderness over abdomen not so intense. Slight tympanites. Vaginal discharge now appears to be natural. Tenderness and swelling on internal surface of the left thigh now seems to be diminishing. No mammary secretion. |
| | 2 | 70 | 30 | | |
| | 3 | 64 | 28 | 2 | |
| | 4 | 64 | 28 | 3 | |
| | | 60 | 24 | 2 | |
| | 6 | 60 | 28 | 2 | |
| | 7 | 60 | 28 | 2 | Bowels moved twice. |
| | 8 | 58 | 28 | | |
| | 9 | 60 | 28 | | |
| | 10 | 56 | 28 | 2 | |
| | 11 | 64 | 32 | 3 | |
| | 12 | 72 | 24 | 4 | |
| | 1 P.M. | 78 | 32 | 6 | |

| | *Hour.* | *Pulse.* | *Resp.* | *Drops.* | |
|---|---|---|---|---|---|
| *March 4th.* | 2 P.M. | 80 | 28 | 8 | |
| | 3 | 80 | 24 | 8 | |
| | 4 | 80 | 30 | 8 | |
| | 5 | 80 | 28 | 8 | Sleeping. |
| | 6 | 60 | 32 | | |
| | 7 | 64 | 24 | 6 | |
| | 8 | 60 | 24 | 2 | |
| | 9 | 60 | 28 | 2 | |
| | 10 | 60 | 24 | 2 | |
| | 11 | 60 | 26 | | |
| | 12 | 58 | 24 | | |
| *March 5th.* | 1 A.M. | 60 | 22 | 3 | She now says she feels much better. Her countenance looks much brighter, and she appears to be much improved in every respect. The tenderness which has been so intense over the abdomen, now is scarcely noticeable. Tympanites very slight. Discharge very scanty, but normal. No mammary secretion. The swelling and tenderness on the internal surface of the thigh, in the course of the veins and lymphatics, has now disappeared altogether. |
| | 2 | 68 | 26 | 4 | Sleeping. |
| | 3 | 60 | 22 | 2 | |
| | 4 | | | | |
| | 5 | | | | |
| | 6 | 70 | 30 | 6 | |
| | 7 | 64 | 24 | 4 | |
| | 8 | 76 | 24 | 6 | |
| | 9 | 76 | 24 | 6 | |
| | 10 | 72 | 28 | 6 | |
| | 11 | 64 | 24 | 3 | |
| | 12 | 68 | 24 | 6 | |
| | 1 P.M. | 64 | 28 | 5 | |
| | 2 | | | | |
| | 3 | 56 | 28 | | |
| | 4 | | | | |
| | 5 | 64 | 24 | 5 | |
| | 6 | | | | |
| | 7 | | | | |
| | 8 | 68 | 26 | 4 | |
| | 9 | | | | |
| | 10 | 72 | 24 | 4 | |
| *March 6th.* | 8 A.M. | 70 | 24 | 6 | Feels well; improvement marked. No tenderness on pressure over abdomen. No tympanites. Discharge still scanty, but normal. Slight mammary secretion. |
| | 11 | 76 | 24 | 4 | |
| | 12 | | | | |
| | 1 P.M. | 72 | 24 | | |
| | 5 | 78 | 28 | 8 | |
| | 6 | | | | |
| | 7 | 76 | 26 | | |
| | 8 | | | | |
| | 9 | | | | |
| | 10 | 72 | 24 | 4 | |
| *March 7th.* | 9 A.M. | 76 | 24 | | She says she feels well and hearty. No tenderness over abdomen. No tympanites. Vaginal discharge healthy. No tenderness or swelling in left femoral region. Appetite good. Bowels regular. |
| *March 8th.* | 10 A.M. | 76 | 24 | | Continues to improve very fast. |

From this time she continued to improve, and in a short time was discharged as well and hearty as she ever was."

The *Memphis Medical Recorder* gives an abstract of an account by Dr. Gorham, in the *Medical Independent*, of the treatment of dysmenorrhœa, by the use of veratrum viride. "He begins two days before the expected period, and gives three drops of the tincture every three hours, with directions to increase one drop at each succeeding dose, and until nausea comes on, and then to reduce the dose again to three or four drops. In one case, the nausea with retching was produced by the seven-drop dose. The next case was vomited freely by the six-drop dose; and the third sickened and vomited at the two-drop dose. The frequency of the pulse was much reduced in all cases, and menstruation in each case came on at a later period by three or four days than the patient expected, but without the least pains, the relief appearing complete."

Hitherto we have introduced, without a break, the testimony in favor of the therapeutical employment of veratrum viride. In doing so, it was deemed necessary to introduce, at the same time, an account of the stages of the disease, and of the prior treatment, as well as of the remedies associated or given in alternation with the veratrum viride in each particular case. Any other fashion of therapeutical history is little better than a mere empirical record, which would be as little calculated to encourage the timid, as to restrain the too sanguine practitioner. The adverse opinions must now be noticed. Among the chief of those who take this side of the question, is Dr. W. A. Brown, of Georgia. This gentleman lays much stress, in the *Nashville Journ. of Med. and Surg.* for October, 1856, on the tendency of this medicine to cause abortion, and, in confirmation of his belief of its deleterious operation in this way, he adduces the certificates of some of his medical friends, viz.: Drs. Fielder, Matthews, and Lamar, of Georgia. Drs. Brown and Fielder go so far as to declare it to be an unsafe remedy in general practice.

Dr. Haughton, of Indiana, in the same journal, for December, 1856, meets the objections of Dr. Brown by positive counter-statements. He says, that, during a period of three years, he has used the veratrum in cases of pregnancy in all its stages, without abortion resulting in any one of them. As regards the safety and efficiency of the use of the remedy, he tells us that he has given it in all ages, from the infant to adult age, and has always obtained the desired results. If the veratrum produces abortion, it must be, as Dr. H. argues, from the nausea or vomiting, which is never necessary when we wish to obtain the expected effects from its administration. The fact, however, cannot be concealed, although it is not mentioned by Dr. H. that nausea and vomiting are quite common and not always preventible effects of the use of this medicine. At the same time, if it be really deleterious in pregnancy, so as to cause abortion, we must needs explain this result by another *modus operandi* than through gastric distress and derangement, which, we repeat, are but symptoms or concomitants of its depressing action on the nervous system, and which are, besides, such common accompaniments of the first months of pregnancy.

Dr. B. T. Newsom, of Georgia, has written a paper (*Nashville Journ. of Medicine and Surgery*, Jan. 1857,) in reply to Dr. Brown, in which he says "most positively and unequivocally, that it [veratrum viride] has been given to and taken by pregnant females so as to have its full sedative effect, and that it has never produced abortion in a single instance within my knowledge, as the following cases will show."—Of these, three were of unmarried females, two of them sisters, the one 16 and the other 14 years of age, who endeavored, but without success, to produce abortion by taking, surreptitiously, large doses (half a teaspoonful repeated in an hour) of the tincture of veratrum viride, which had been left in the house for the use of their married sister. All these cases are to the point, and we would gladly repeat the record of them did space permit.

Dr. Newsom's narrative of the cases of the two unmarried sisters is preceded by a brief statement to show, what it was necessary for us to know, how the veratrum had been used for a criminal purpose without his knowledge or participation in any way. As mentioned above, the tincture had been left with the married sister for her own use under circumstances indicated at the time. The vial containing it had been abstracted afterwards, first by one and then by the other of the two unmarried sisters, who had learned from Dr. N.'s patient the opinion communicated to her by him, that some physicians believed in its power of producing abortion.

The fourth case related by Dr. Newsom was of a married female, pregnant four months, who, on account of her husband's misconduct, wished to produce abortion. With this view she took two doses of the tincture of veratrum viride, each of thirty drops. She was made alarmingly sick in consequence, but went to her full time.

Touching the question of safety of the use of veratrum viride, Dr. Newsom says, that, since the year 1850, he has given it in numbers of almost every variety of inflammatory affections, *gastritis* excepted, and that he has never seen any *ill effects* from its use. But this gentleman is far from regarding veratrum in the light of a panacea, to be used to the exclusion of other and milder agents, which, in certain cases of inflammatory disease, would succeed equally well. Nor would he overlook the necessity of its being given with caution, and of its effects being carefully watched, "just as should be the case with every other remedial agent of like power; due regard being always had to the nature of the disease, condition, situation, and surrounding circumstances of the patient at the time." In this connection it may be deemed worth while to repeat a fact mentioned by Dr. Taliaferro. It is, of a physician of Atlanta having swallowed, through mistake, an ounce of the tincture at one draught. The consequent disturbances only amounted to extreme nausea and vomiting, and some difficulty in breathing. We are not told whether any attempt was made to relieve, by other means, the stomach from this unaccustomed and undesired dose.

After this survey of the different uses to which veratrum viride has been put in the treatment of disease, it is easy to see that much yet remains to be known concerning its therapeutical capabilities. So far, its use would seem to have been confined to febrile and inflammatory diseases; but, if we can attach any importance to the analogy which this substance bears to the class of sedatives belonging to the vegetable kingdom, we must believe that its greater range and variety of application will be found in subacute and chronic affections, in which the nervous system and nutrition are implicated,—as in the neuroses; and in scrofula and the secondary stages of syphilis, as well as in chronic rheumatism and gout. May it not deserve, in various cardiac affections, especially in hypertrophy, the epithet applied to digitalis of its being "the opium of the heart?" Beyond the sedative effects of veratrum viride we have very little accurate knowledge of its action on the organism. Experiments, both physiological and clinical, are wanting to show to what extent it acts as a diaphoretic or expectorant, although these properties are claimed for it by its eulogists. The natural tendency of a febrile paroxysm to end in sweating, and of pneumonia to relieve itself, after a few days, by copious expectoration, should make us slow to admit the efficacy of an agent as a diaphoretic or an expectorant in these diseases, merely because diaphoresis and expectoration follow its use. The operation of veratrum viride in either of these ways has yet to be tested by its trial in a much wider circle of disease, and with more guards against fallacies than have yet been had recourse to. We have heard nothing of its action on the kidneys; and in this respect it differs greatly from digitalis. As an adjuvant and corrigendum to purgatives, especially those of the resinous and drastic order, much good might be expected from veratrum viride, which in this, as well as in some other particulars, will be found to resemble hyoscyamus.

*Gelseminum Sempervirens—Yellow-Jessamine.*—Within the last year, additional knowledge has been gained of the medicinal virtues of another indigenous plant, the name of which heads this paragraph. For this advance we are chiefly indebted to Dr. J. A. Mayes, who, in a paper read before the "South Carolina Medical Association," and which was published in the *Charleston Medical Journal and Review* for March, 1857, has collected the observations and experience of others, in addition to his own, on the subject. In his introductory remarks, Dr. Mayes says:—"The well-known poisonous properties of the flowers of the gelseminum or yellow-jessamine would seem to indicate that the plant is possessed of powers that, if properly managed, might be advantageously employed in the treatment of many diseases." The author modestly tells us, that his essay must necessarily partake of the character of a compilation, and be dependent for its substance principally upon copious extracts from the writings of others. It is this very character of compilation which makes Dr. M.'s paper more available to our present purpose, and, in this sense, more valuable.

Dr. Cleveland, of Cincinnati, is quoted for the history of the discovery of the virtues of the yellow-jessamine. A planter, suffering under bilious fever took, by mistake, an infusion of the root of this plant, and was cured, although for a time he lost all muscular power. Dr. Batchelor (*Iowa Med. Journ.*) gives a similar account of its introduction into general practice.

The mode of preparation and administration of the gelseminum are next to be considered. It has been little used except in the form of tincture, which is thus prepared by Dr. Batchelor:—"A bottle is loosely filled with the bark of the fresh root; equal parts of whisky and water are added; and the bark is macerated for fourteen days; twenty to sixty drops of this infusion may be used at a dose, alone or combined with quinine." Dr. Mayes prefers the following formula as the one after which he prepares the tincture, and as that which is understood to be the standard of the article in the cases which are described by himself:—Four ounces of the fresh root, chipped small, to one pint of diluted alcohol; macerate for fourteen days. Of this tincture he gives to adults from twenty to fifty drops, repeated as frequently as circumstances may require. The root would seem to contain a resinoid principle soluble in strong alcohol, the ingestion of which has, we are told by Dr. Cleveland, given rise to some deaths in New Orleans. Hence, he thinks that the best menstruum is diluted alcohol, or even water, in which "the sedative property is fully soluble." The dose of the saturated tincture which he employs, is from three to thirty drops: he deems it to be "much safer and better to give small doses, frequently repeated, than to risk too large a dose of a powerful sedative medicine." On this point, Dr. Nash, of Norfolk, Va., says, that the tincture of the root, the preparation used, has a characteristic odor; and that it may be given in doses, for adults, of from ten to fifty drops, in a little water; and even one or two teaspoonsful have been administered, as it varies in strength, owing to there being no fixed standard for its preparation.

Dr. Cleveland expresses his unqualified faith in the sedative properties of the gelseminum through its operation on the spinal nerves, and, in a less degree, on the eighth pair and branches of the sympathetic distributed to the heart and lungs, by which it diminishes the force and frequency of the pulse and lowers the rate of respiration. Dr. Branch, of South Carolina, (*Charleston Med. Journ.*) remarks, that this article is "narcotic in an intense degree, but it possesses other powers—perhaps deobstruent and tonic—as well as narcotic." Dr. Batchelor gives a more extended idea of its therapeutical powers, when telling us that it is narcotic, nervine, antispasmodic, and sedative, under which last light he considers it to be superior to digitalis, sanguinaria, or even veratrum viride. It possesses the advantages of being by no means unpleasant to the taste and of not producing nausea. "In full doses it produces a sort of intoxication, languor, dizziness, double vision, inability to raise the eyelids, and, in an overdose, complete muscular prostration." Dr. Nash, of Norfolk, looks upon it "as narcotic, antispasmodic, and sedative; seemingly spending its influence, chiefly upon the sensory ganglia, the spinal cord, and voluntary muscles, and leaving unaffected the intellectual faculties. It reduces the circulation and promotes perspiration and the secretions generally, without causing nausea, vomiting, or purging, and has been given in every stage of febrile diseases." We do not see on what grounds Dr. Nash claims narcotic properties for the gelseminum, as it does not act on the brain, by inducing either sleep or stupefaction; both of which, in greater or less degree, result from the operation of a narcotic, which, moreover, excites, before it produces its sedative impression; a course of things not ensuing, as far as we can learn, on the use of the article in question. Dr. N. describes the physiological effects of the medicine to be, dimness of vision, double vision, inability to open the eyelids, stiffness of the jaws, general muscular debility and complete prostration. These, he adds, soon pass off and leave the patient comfortable and refreshed. We know not how to reconcile the "stiffness of the jaws," in the above enumeration, with the other symptoms of muscular relaxation.

Dr. Mayes assures us that he has never, in a single instance, been disappointed in obtaining a direct sedative action from the use of the gelseminum; "the patient being speedily quieted, although he may have been excessively agitated previous to its administration. Under its influence, restlessness is soon succeeded by calm repose, and the excited, frequent pulse tempers down to tranquillity. These favorable impressions must be secured, however, by a frequent repetition of the dose, as its effects are not very durable, wearing off in two or three hours. It will be found necessary to administer the medicine in doses of from twenty to fifty drops, according to the severity of the symptoms, every two or three hours, until, under the influence of more radical remedies, the disease has been permanently controlled." Dr. M.'s desire, in thus advocating the use of this medicine, is "to bring it into notice as an admirable agent for controlling irregular nervous action and bringing about in the system a state of repose favorable for obtaining the full action of other and more radical treatment." In language like this we recognize a man of good sense and observation, who, in his love of novelty, does not forget old and tried resources, and who knows that the suppression of a particular symptom is not a removal of the disease. We can refrigerate the most hot and burning skin in fever down to an almost icy coldness, but without getting control of the fever, or of the morbidly-excited and heat-generating apparatus in the organism.

In comparing gelseminum with veratrum viride, Dr. Mayes admits the greater power of the latter; but from this very circumstance he thinks that it is not so generally applicable in practice, and that it is too energetic in its action to be entrusted in the hands of even the most intelligent nurses. In his comparison of the two he mentions their relative efficacy in a case of severe pneumonia, in which the gelseminum failed to reduce the pulse under 120 during a few days' use of the article, while in the short period of a few hours it was brought down to 80 by the veratrum viride. He concludes by saying:—"From the result in this case, I am persuaded that gelseminum cannot supersede entirely the veratrum viride, but, being less energetic, it can be more safely used in general."

Dr. Cleveland remarks of the veratrum, that it sometimes produces such an amount of nausea, and even vomiting, as to preclude the farther use of the remedy; it also, not unfrequently, induces such free catharsis as to injure the patient if its use be continued. Among the numerous accounts of its effects, this is the first one in which a free action on the bowels is attributed to this article. The gelseminum, on the other hand, continues Dr. C., "while it proves a most powerful sedative to the nerves supplying the muscles of locomotion, as in the case of the planter who first took it, never seems to produce the slightest effect on the brain, or in the least degree to derange the stomach or bowels. It *does* lessen the pulse and the frequency of respiration; and while it produces a wonderful relaxation of all the muscles, it relieves all sense of pain, by acting on the nerves of general sense in a manner similar to aconite."

On the first discovery of gelseminum it was announced to be a speedy cure for intermittent fever, and it has been very largely used in the Western States in the form of various secret nostrums. Dr. Nash, already quoted, puts a high value on its febrifuge powers. He has used it in every case of idiopathic fever that has fallen under his charge for the six months preceding the date of his writing, with perfect impunity, and with the most desirable results; neither age nor sex interfering with its exhibition. He does not, however, rely upon it solely in all cases, especially those of a high grade; but he thinks it entitled to rank as a *coefficient* with *quinine* in fevers. In this respect he stops short of others farther South, who speak of it as a specific in this class of diseases.

Dr. Mayes, in summing up his own opinion of gelseminum, deduced from actual observations of its effects, thinks it is a most valuable adjuvant to other treatment, in all cases where high arterial action exists, and where, as in the case of injuries, it is desirable to lessen the irritability of the nervous system: in hysterical affections its value cannot be too highly estimated.

Dr. John Douglass, of South Carolina, in a letter to Dr. F. P. Porcher, (*Charleston Med. Journ. and Review*, July, 1857,) gives his experience, during a period of thirty years, of the curative powers of the yellow-jessamine (*gelseminum sempervirens*) in gonorrhœa. He uses a tincture made of diluted alcohol, as follows:—A small handful of the root is put into a common junk bottle of whisky, and after a day or two days' infusion, the patient is ordered a tablespoonful of the tincture night and morning. Temporary disturbance of vision sometimes follows the use of the medicine.

Dr. Elliott, in his Botany of South Carolina and Georgia, says of the gelseminum, that the flowers, root, and the whole plant are narcotic; and that a saturated tincture had been long used with marked success in rheumatism.

*Aconitum Napellus—Aconite.*—Dr. Edward B. Stevens, of Cincinnati, reports (*Cincinnati Medical Observer*, Oct. 1857,) his success, confirmatory of the experience of others, in the use of the aconite, for the cure or relief of "almost the entire range of neuralgic affections, and of those obscure complications, of rheumatism and neuralgia, in which there is freedom from local or constitutional trouble, independent of nervous derangement."

In a case of neuralgia "supposed to be a result of previous attacks of miasmatic disease," and which was treated by the use of quinine and other remedies with but temporary relief, Dr. Stevens prescribed a mixture of the tincture of aconite and tincture of cimicifuga, which gave entire relief to his patient. The proportion was—℞. Tinct. rad. aconit. ʒi.; Tinct. cimicifuga, ℥ij.—M. Dose, a teaspoonful every four hours. Three doses were sufficient to procure the desired relief. Ten months had elapsed without a return of the disease.

A case of neuralgic rheumatism of the arm, of peculiar obstinacy, after having been intractable to all remedies, yielded to the aconite. In the above formula, each dose should be equivalent to about four drops of the tincture, although, in fact, this latter gives somewhat more than sixty drops to the drachm. In this dose, of four drops, Dr. Stevens has "never seen any effects sufficiently marked or evident to occasion alarm."

Dr. S. has not used this article in acute rheumatism; but in chronic rheumatic pains, particularly in old people, he has derived excellent effects from it. In dysmenorrhœa, or neuralgia associated with uterine derangement at or subsequent to the catamenial period, entire relief, followed by refreshing sleep, has been obtained by the administration of the aconite.

In the diminished and sometimes abolished sensibility and voluntary motion to which aconite gives rise, we find similarity of effects to those produced by veratrum viride and gelseminum.

*Valerianate of Ammonia.*—Dr. W. Hay, of Chicago, (*North-Western Med. and Surg. Journ.*, Oct. 1857,) relates his experience of the valerianate of ammonia in a case of neuralgia. The subject was a lady laboring under incipient tuberculosis and anæmia, who was seized (June 6th, 1857,) with severe neuralgic pains in the right temple, extending over the entire half of the head and face. The pains were mitigated by the local application of chloroform, but the relief was not complete until after the lapse of five or six hours. During the six following days the patient still suffered. On the 19th June, Dr. Hay directed the following mixture:—R. Tinct. val. ammon. ʒvi.; Tinct. opii, ʒij.—M. One teaspoonful to be taken every three hours; local applications to be continued. This treatment was continued for several days, but without any sensible effect, except great irritability of the stomach, with excessive nausea and vomiting. In place of the valerianate, the tincture of aconite was prescribed in doses of five drops every two hours, and also its application externally; but although its use was persisted in for twenty-four hours, no benefit ensued. On the contrary, the paroxysms were greatly lengthened, so as to require a return to the application of the chloroform externally, and its use by inhalation. July 4th.—The pain still continuing with slight intermissions, the precipitated subcarbonate of iron was taken every four hours, alternated with four grains of the subnitrate of bismuth. The nausea was, by this means, relieved and the pain ameliorated for two or three hours only, after which the pain returned with increased violence. July 6th.—Fowler's solution six drops, and a drachm of the tincture of Cannabis Indica every six hours were equally unavailing. July 7th.—Recourse was again had to the valerianate of ammonia in a deliquescent state, of which one drachm was dissolved in an ounce of water,—dose, a tablespoonful. A marked decrease of pain and augmented force with diminished frequency of the pulse, followed the administration of one dose at 7 P.M. After taking the second dose at 10 P.M. the patient sank into a quiet sleep, which lasted till morning. July 8th.—The same treatment was continued this evening as on the last, but although the pain was much diminished it had not entirely gone. The same prescription was continued on the two following days; but on the 14th, as the salt seemed to have lost its controlling power over the disease, and the duplication of the dose caused distressing dyspnœa and headache, without relief of pain, precipitated subcarbonate of iron in the dose of half a drachm was prescribed. This was followed by immediate and permanent relief from pain, which did not return. The treatment was persisted in for a few days, as a precautionary measure.

Another trial of the valerianate in the same case, on the occasion of a return of the disease, a month afterwards, was attended with more permanently beneficial results. Other remedies had been tried in vain. A temporary suspension of the "pain had been procured by the inhalation of chloroform." One grain of the crystallized salt of the valerianate of ammonia, dissolved in a teaspoonful of water, gave almost immediate relief; and in fifteen minutes the patient was asleep. The relief has been permanent.

Dr. Hay attributes his success, in the last trial of the valerianate of ammonia, to the greater strength of the article. The specimen first used had absorbed so much water as to become perfectly deliquescent.

The formula used by M. Declat, to whom we are indebted for a knowledge of the efficacy of the valerianate in neuralgia, is as follows:—Distilled Water, two drachms; Valerianic Acid, one drachm; Subcarbonate of Ammonia, enough to neutralize the acid; then add Alcoholic Extract of Valerian, two scruples. The dose was, on the first night, a teaspoonful, and on the following day, two teaspoonsful. The dose was raised to a dessertspoonful night and morning.

The *Southern Med. and Surg. Journ.* tells of satisfactory results in two cases of obstinate temporal and facial neuralgia. The "*telling* dose" in the first, was three grains of the salts of the valerianate of ammonia in solution, and in the other, four grains of the same—each three times a day. The editor thinks that large abatement may be made from the extravagant eulogies in favor of this remedy in some quarters. The remark is applicable to all new remedies.

As valerian itself is usually spoken of as a stimulant, objection may be made to our placing the valerianate under the head of sedatives. Its speedily soothing operation and anæsthetic effect are so obvious, that they justify, in our mind, the place which we have assigned to the salt. We may add, as corroborative evidence, that M. Declat was led to the employment of the valerianate of ammonia in neuralgia, by the relief which he procured for himself from its use, when suffering from frequent headaches, which he attributed to a severe attack of meningitis. He experienced all the sedative effects of opium from the remedy, without the cerebral disorders with which he was always afflicted when he had recourse to the former.

*Bi-sulphuret of Carbon.*—Mr. James Schiell, of St. Louis, (*St. Louis Med. and Surg. Journ.*, Jan. 1857,) describes the mode of application, and some of the therapeutical uses of this substance. He directs that, of a mixture composed of equal parts of the bi-sulphuret and alcohol, a little shall be poured on a tuft of cotton, which is to be rubbed "pitilessly over the affected part four, five, or six seconds. A strong, burning sensation follows, lasting only a few seconds, with the cessation of which ceases also the suffering of the patient. Sometimes a second or even a third application, at intervals of two or three minutes, is necessary, and the pains will disappear as if the suffering part had been touched by a magic rod." In two cases of bilious colic, after a strong dose of calomel had been given, friction over the abdomen, with the mixture as above, (bi-sulphuret of carbon and alcohol) removed the pain in a few seconds. In addition to the treatment just mentioned, an injection, containing half a teacupful of glycerine, was administered.

*Strychnia in Sciatica.*—Dr. O. C. Gibbs, of Frewsburg, Chatauque County, New York, (*American Med. Monthly*, September, 1857,) relates an obstinate case of sciatica which had resisted colchicum, ammoniated tincture of guiacum, quinine and morphia, oil of turpentine, tincture of cimicifuga, iodide of potassium, Dover's powder, blisters over the great trochanter, and the endermic application of morphia, cups over the same part, and colomel to touch slightly the gums. For some time the patient, despairing of relief, had been in the habit of using opium, which was given at the discretion of his wife, as the only means of assuaging the pain. Dr. Gibbs describes his mode of using the strychnia, and its success, in the following terms:—

"We now took two grains of strychnia, in crystals, and put it with two ounces of water, slightly acidulating the water with sulphuric acid. We also took four grains of podophyllin and five of sulphate of morphine, rubbing them up with sugar, and dividing them into twenty powders. We ordered thirty drops of the solution of strychnia, also one of the powders, to be taken three times a day. The patient had no severe paroxysm of pain after this, and within three weeks from the time of commencing the strychnia, he went down the Allegheny and Ohio Rivers as a pilot on a lumber raft, and up to the present time he remains free from a return of the affection." The author has omitted to say when the successful treatment was begun, and at what date he wrote the account of the case. He took charge of his patient March 6th, 1857. We are left to gather from the narrative, that the trial of the long list of remedies above mentioned lasted about three or four weeks before recourse was had to strychnia. Each dose of the solution of this substance, as directed by Dr. Gibbs, was equivalent to the sixteenth of a grain.

*Veratria and Morphia in Incontinence of Urine.*—Dr. Thos. Kennard, Assistant Physician to Blackwell's Island Hospital, New York, reports (*American Journ. Med. Sciences*, Jan. 1857,) three cases of incontinence of urine, cured by the external application of veratria and morphia. His formula is:—℞. Morph. Sulphat. Veratr. āā. gr. x.; Axungiae, ℥j.; ft. Ung. The perinæum is to be rubbed with this ointment three times a day.

*Etherization in Convulsions.*—Dr. N. J. Knight, of Somerville, Massachusetts, (*Boston Med. and Surg. Journ.*, February, 1857,) records a case of puerperal convulsions, which followed abortion. "Bleeding, a cathartic, cold to the head, sinapisms to neck, legs, etc. did not prevent the recurrence of three or more convulsions in less than ten hours." Dr. Knight then began to administer sulphuric ether, (by inhalation,) and although no more convulsions occurred, it was not until the end of forty-eight hours that the nerves became so calm as to allow the ether to be omitted altogether. Ten days from the first attack, the lady was delivered of a seven months' child, which had evidently been dead from the time of the first convulsion." We are not told how often the inhalations were repeated, nor how much ether was used, nor the degree to which the etherization was carried in this case.

Dr. Knight mentions another case of convulsions in a married lady, which were promptly and effectually removed by the inhalation of ether. He considers "ether really the only safe and efficient remedy, for convulsions of *teething children* or adults, now known to the profession."

Dr. Ellis (*Boston Med. and Surg. Journ.*, Jan. 1857,) reports the case of a woman, aged 35 years, who, when eight months pregnant, was seized with convulsions, which were so continued and severe that the case was deemed hopeless, when Dr. Storer recommended the administration of ether by inhalation. This was had recourse to for about two hours, after which time there were no convulsions. The patient recovered her consciousness, but sank, after some days, under gangrene of the lungs. A dead child was born not long after the etherization.

Dr. Wm. Dickey, of Centreville, Indiana, reports a case (*Western Lancet*, May, 1857,) of puerperal convulsions of a very violent character following the first labor, which ceased on the administration of the chloroform, although the coma lasted two days afterwards.

A case of violent convulsions accompanying and immediately following difficult labor, in which turning was practiced, and after the birth of the child, chloroform was given by inhalation with entire success, is recorded by Dr. Oliver, among *Extracts from the Records of the "Boston Society for Medical Improvement."* (See *Boston Med. and Surg. Journ.*, Feb. 1857.) The patient, who became unconscious after the first convulsion, remained so for some days; the recovery was very gradual, and not without occasionally alarming symptoms.

Dr. H. R. Storer, of Boston, strongly recommends the use of anæsthetic agents in cases of infantile convulsions, where no actual organic disease of the brain exists. He reported (*Boston Journ., ut supra*) a case where a girl, 12 years old, who had never menstruated, was taken with convulsions. The first night of her illness she was relieved by the ordinary course of warm bath, emetics, etc. The convulsions returned on the second night, when, having in vain employed the ordinary treatment, and the patient evidently failing, Dr. S. administered chloroform. The single convulsions were at once stopped by a short inhalation; and, finding its good effects thus manifested, he kept her well under its influence for half an hour, after which there was no further return of convulsions.

Dr. Casey, of Middletown, Conn., adds his testimony to that of others in favor of anæsthesia by chloroform inhalation. He has used the remedy in various forms of convulsions—puerperal, infantile, epileptic, and hysterical—and he has scarcely ever failed to find it beneficial.

*Tobacco in Erysipelas.*—Dr. John G. Stephenson, of Terre Haute, Indiana, desires to call the attention of the profession (*Western Lancet*, May, 1857,) to the treatment of erysipelas which has proved so beneficial in his practice, that the use of it has become, with him, a matter of routine. "The treatment is simply the covering of the inflamed surface with wet tobacco leaves, (such as are to be had in any cigar shop,) which are permitted to remain until much nausea is produced." Dr. Stephenson, while he admits that the excessive and distressing nausea produced by the internal use of tobacco prevents its administration by the stomach, is willing to believe in its power and safety when cautiously applied to a cutaneous surface, as a remedy for local inflammation.

We must, however, be aware of the difficulty, not to say impossibility, of determining, after no matter how many trials, the extent of surface to be covered, and of absorption produced by this application of the tobacco, so as to procure the desired amount of sedative effect and accompanying nausea. The difference between the internal and external use of tobacco is only one of degree; and in both instances there is uncertainty and risk of alarming, if not fatal results. Hence the great caution always practiced in the administration of this plant as a therapeutic agent—a caution which is especially called for in cases of erysipelas in old subjects with broken-down constitutions and slight powers of reaction.

In one of the cases related by Dr. Stephenson, the patient was pregnant about five months. She soon got well under treatment. This consisted in the use of calomel, followed by saline purgatives, Dover's powder, sulphate of cinchona, and the local use of tobacco. In another case of a person aged 17 years, slightly chlorotic, in which tincture of the chloride of iron internally and tincture of iodine externally failed to prevent the extension of the inflammation of the leg from above the ankle to above the knee, the application of the wet tobacco leaves soon produced extreme nausea and prostration, followed, after several other renewals of the same topical treatment, by a complete removal of the inflammation.

*Chloroform Inhalations in Delirium Tremens.*—Dr. F. M. Garrett, of Tarborough, North Carolina, gives (*American Journ. of Med. Sci.*, April, 1857,) a "report of five cases of delirium tremens treated by the inhalation of chloroform." The remedy was had recourse to after other means had failed to produce sleep, or to quiet the patient. In the first case, a man aged 34 years, to whom brandy and morphia had been given without sleep having been procured for thirty hours, ℥ij. of chloroform "were inhaled, when the patient was breathing stertorously. The administration occupied half an hour. At its commencement the pulse was 144; when under its influence, 128. Respirations nine per minute. There were several convulsive movements during the inspiration. He slept soundly for about an hour, when he awoke, but almost immediately slept again. This occurred two or three times during the night. 20th.—The patient expresses himself as greatly improved, and feels able to write. He is perfectly rational. No treatment except good diet and quiet. From this time he improved, and was discharged the 24th" (of September, 1852,) eight days after his reception into the hospital.

The second case was of a man aged 40 years, of good constitution, and generally enjoying good health, but of intemperate habits for the last five or six years. Brandy and opium gave partial repose the first night of his admission; but after this, on the second day, the administration of sulphate of morphia and tartrate of antimony had no effect. The patient is only kept in bed by force; trembles violently, and is bathed in perspiration. Eyes suffused. Pulse 120. "A drachm of chloroform was poured on a towel and applied to the nose—the pulse having been closely watched by an assistant. After inhaling about two minutes, a slight tremor of the lower extremities was noticed. No change in the pulse. The towel was removed for about five minutes, when, the tremor having ceased, another drachm of the chloroform was administered. In a few minutes patient was sleeping soundly. Pupils, before widely dilated, now contracted. Slept about one-quarter of an hour, when he awoke. Two drachms more of chloroform were administered; the towel not applied closely to the nose. In ten minutes he fell asleep, and slept the remainder of the night—13th (November, 1851; three days in the hospital.) 10 A.M., patient entirely rational; complains only of weakness; pulse 85, tolerably full; skin comfortable; ordered nourishing diet. No further treatment necessary."

In the third case, the patient was 30 years of age; had been very intemperate for the last eight years. Opium was ordered during two successive days and nights. On the second night seven grains had been taken, but without sleep being produced. Patient wild and disorderly; had terrifying apparitions. On the third night seven grains of opium were taken, but without sleep following. At 10 A.M., he was put under the soporific influence of chloroform, to effect which two and a half ounces were administered. "He yielded to its action several times before the quantity had been taken, but would wake up immediately and become riotous. While under its influence, the pupils were strongly contracted, dilating when he aroused, only to contract again when he yielded to the influence of chloroform. He awoke after five hours sleep, and took a little beef tea. His condition little improved. Ordered tinct. opii in forty-minim doses, with beer, every two hours till sleep is produced—11th [October,

1851, the 4th day of delirium.] He took 60 minims of tinct. opii, which effected a long, quiet sleep. He is, this morning, quite rational, and has an appetite. Ordered two ounces of beef tea, for which he craved, and a pint of beer daily, in addition to the diet of the house. 12th. Much improved. Discontinued opium and brandy, and gave tonic mixture. 14th. Transferred from ward, cured."

The fourth case was of a man aged 45 years, who had been drinking excessively for some time previously, and suffering under dysentery for a week. Has slept none for several nights. On the second day after his admission into the hospital, which took place September 17th, 1850, chloroform was administered. "At the beginning of the inhalation, the pulse was 130, and he was perspiring moderately. He yielded to its influence very quickly, but the respirations became so slow, that he had to be roused from his stupor, and made to breathe. This followed every attempt to bring him under its influence, and he was consequently only partially etherized. Some convulsive movements occurred during its administration. He slept only a short time, but the wildness was gone, and, at intervals through the night, he slept awhile. When under the influence of chloroform, the pupils contracted to a point, and were so immediately when awaked. 20th. Patient is worse this morning, being comatose. Treatment was continued through the day, but patient continued to sink, and died at 7 P.M. *Autopsy seventeen hours after death.*—Intense arachnitis, with serum effused under the arachnoid membrane. Lymph over the surface; ventricles normal; some serum at base of brain."

The subject of the fifth case was 52 years of age; admitted Sept. 20th, 1851. He had been drinking excessively for some time past, and had dysentery for three weeks. Was nervous, and had been delirious during the three past nights. Pulse regular, and not too frequent. Half an ounce of brandy and ʒj. tinct. opii were ordered every hour till sleep was produced. 21st. Patient had slept about four hours during the night, and the dysentery is checked. 22d. Had slept none for thirty-six hours, and was so wild through the past night, that it was necessary to confine him to bed. Treatment, as above, continued without intermission, but without good effect, and by night the dose of the tinct. of opii was doubled. This was continued till 1 A.M., when it was deemed necessary to administer chloroform. "Pulse at beginning, 128. Patient furious, and sweating freely. When a small quantity had been inhaled, convulsions of the most severe kind came on, causing for awhile the suspension of the chloroform. These occurred several times during the inhalation, and generally on the application of fresh chloroform. In about half an hour the patient was brought fully under its influence. Pulse 120. Otherwise, about the beginning of the inhalation, he slept about three hours, when he awoke, and the chloroform was again administered with the same results as before. He slept till morning. 23d. Felt pretty comfortable through the day, but became wild at night, although the treatment before mentioned was continued only when he was asleep. At 11 A.M., as the patient was unable to sleep, chloroform was again administered. Pulse 90, tolerably strong. During the inhalation he suddenly became furious, and attempted to strike those around him, and soon afterwards, convulsions of the kind above named seized him. Three of these occurred. In about twenty minutes the full effect was produced, and the patient slept till morning. Pulse about as at the beginning. 25th. Patient is improving. He sleeps well, and complains only of debility. Ordered a nourishing diet, and a moderate amount of stimulus. 28th. Patient is convalescent."

In three of these cases Professor Metcalfe was the visiting physician, and in two, (the second and the third,) Dr. McCready. The house physicians were Dr. Elliott, in the first, fourth, and fifth cases, and Dr. Olmstead in the second case. No name is designated in the third one.

In the same number of the Journal, we read an account by Dr. W. M. Chamberlain, of Astoria, Long Island, of eight cases of delirium tremens, in which chloroform was inhaled "as the '*ultima ratio medendi*,'" with the effect of procuring sleep and tranquillity, and restoring the patient. In one case pneumonia followed, and proved fatal; in another the patient, after restoration to consciousness, sank exhausted. In one case the respiration was suddenly suspended upon becoming stertorous; in another asphyxia was produced suddenly, "but the disease was as suddenly vanquished." In a third the asphyxia came on shortly after inhalation had been stopped; while in another, both asphyxia and syncope followed the inhalation. All these were restored by means of artificial respiration.

*Stramonium in Convulsions.*—Dr. R. H. Salter, of Boston, relates his experience in the use of stramonium both in puerperal and common convulsions. (*Boston Med. and Surg. Journal*, March, 1857.) In reference to the first of these, he passes in review some of the modes of treatment which are had recourse to. We omit this enumeration and his criticisms on the occasion, which will be noticed more appropriately in the Report on Obstetric Medicine, in a future number of this Journal.

Of the six cases of puerperal convulsions detailed by Dr. S., five were of females in their first labor; the subject of one was attacked in her fifth labor. In the first case, the convulsions occurred on the eleventh day after delivery, and they left behind them permanent partial paralysis of the right side of the body. Age, 26 years. Half an ounce of the tincture of stramonium was given at a single dose. Dilatation of the pupils ensued in forty minutes, and the convulsions ceased. In the second patient, age 20 years, the convulsions came on just as the head was passing the superior strait. She took half an ounce of tincture of stramonium, with tincture of ergot—quantity of the latter not stated. Convulsions returned but once afterwards, and a healthy living child was born. In the third case the labor was slow, and the convulsions assumed a tetanic character. Relief procured in the same way as in the preceding case. The fourth patient had slight uterine hemorrhage for a week before the coming on of labor. A very large blood-letting failed to abate the violence of the convulsions, which were arrested completely by the tincture of stramonium, in a dose of ten drachms, given as an enema; the patient being unable to swallow. She was delivered of a small dead child. In the fifth case, that of the fifth labor, convulsions oocurred eight or nine hours after labor had set in. The convulsions ceased, and the patient became tranquil in half an hour after the administration of the tincture of stramonium. The sixth case was that of an unmarried female, aged 19 years. The fits came on at the time the head was passing the superior strait; they ceased after the administration of half an ounce of the tincture of stramonium, with ergot—quantity of the latter not stated. Five hours after the birth of the child, pain and convulsions supervened, which were arrested by a dose of five drachms of the tincture of stramonium.

Dr. Salter, in concluding his history of these cases, which we have abbreviated, remarks, "that the common and hysterical forms of puerperal convulsions will tolerate, and, for the most part, do well, under almost any rational and judicious treatment. Still, in these forms, I consider the treatment with stramonium as far preferable. It is in the epileptic form, the most frightful and formidable of all puerperal convulsions, that the common treatment is most likely to fail; and if, perchance, it should not fail, it is replete with peril, not only immediate, to life, but also to the future health of the individual. It is in this form that the comparative value and power of stramonium to control spasmodic action is most strongly exhibited."

Dr. Salter, although an enemy to large bleeding, practiced on these occasions, admits that there are cases in which the inflammatory excitement is great and manifest, in which a moderate bleeding should be premised as a *preparatory* measure. Ergot and lupuline will also be found necessary adjuvants, and other articles which might be mentioned; but in no case is any one of them a remedy *per se* for convulsions.

Dr. Salter describes two cases, one a male, aged 17 years, the other a single female, aged 24 years, who had suffered from convulsions, and to both of whom he gave tincture of stramonium with success. In the first case, he directed the tincture to be taken in one-drachm doses every fifteen minutes, until four or five doses were taken. On a repetition of this course, the medicine was directed to be continued at intervals of four hours after the fourth dose, as above. The female had had convulsions. The boy had no more fits after the second day from beginning with the stramonium. The female had had convulsions, simulating tetanus, for thirty-six hours. Dr. Salter ordered the use of the tincture of stramonium in half-ounce doses. If the first did not relieve the patient within half or three-quarters of an hour, the same quantity to be repeated. "In less than half an hour after the medicine was given the convulsive action ceased, and the woman became perfectly quiet, fell asleep, and did not awake until next morning, when she rose, feeling perfectly well, experiencing no inconvenience whatever from the disease or the medicine."

Dr. Salter makes an apposite, and, at the same time, a practical remark, in conclusion. It is that, in his belief, many of the cases reported as tetanus are only counterfeits of this disease.

*Cannabis Indica—Haschisch—Indian Hemp*—Called also *Gungha*, *Bung*, etc.—Dr. John Bell, of Derry, New Hampshire, has a paper, in the *Boston Med. and Surg. Journ.*, April, 1857, on the effects of this plant, chiefly in a psychical point of view. He describes the manner in which he was affected when he put himself under its influence. Room is not allowed us to give even an analytical notice of his opinions and observations on this interesting theme, and we must be content to note a few passages. Among these, is one on the resemblance between a state of mind produced by the *Haschisch* and that occurring in Mania—a resemblance noticed both by M. Moreau and Dr. Bell:—"In both states there is the same excitement and abruptness of manner, the same rapidity and incoherence of thought, the same false convictions and lesions of the affective faculties."—"There is no error of judgment, no delusion or lesion of the will or moral faculties, which is seen in the former state, [Mania,] but what might take its rise in the latter." Dr. Bell describes the series of mental phenomena which were produced on himself by this substance:—"Amid all the strange vagaries of the *Haschisch*, the mind preserves the power of taking cognizance of its condition, and, to a certain extent, of analyzing its operations. The memory of everything said and done is nearly perfect; but of the multitude of thoughts, only those making a more than commonly distinct impression are preserved." He thinks that considerable light might be thrown on insanity, and especially delusions, by watching and analyzing the effects of the Indian hemp, which we may take as a picture of the mind when under another and more enduring series of morbid impressions.

Speaking of its operation on the brain, in reference to the psychical phenomena produced in consequence, Dr. Bell thinks that *five* grains is the smallest quantity from which any perceptible effects are to be expected, and generally more will be required. This opinion coincides with our own experience of the drug (the extract of *Cannabis*) when we have administered it for the alleviation of pain, as in neuralgia, and to procure sleep in delirium tremens.

Dr. A. Bryant Clarke, of Holyoke, (*Bost. Med. and Surg. Journ.*, May, 1857,) tells us, in reference to Dr. Bell's statement of the dose of the Cannabis Indica required to produce the desired effect, that a pill of the English extract, in the quantity of two grains and a half, given to a maniacal patient, who had previously taken the medicine in two-grain doses, produced very marked effects. On visiting his patient, two or three hours after she had taken the pill, Dr. Clarke found her sitting up, and more rational and quiet than she had been for weeks:—"The attendant described her as apparently fainting, with respiration slow and regular, a blue and dusky state of the skin, blood settled under the finger nails, and said they had with difficulty kept her alive." Dr. Clarke being incredulous of the effects attributed to the medicine, swallowed one of the pills, soon after a hearty dinner. Within an hour he began to feel its peculiar effects, such as are so well described by Dr. Bell. Dr. Clarke's left arm was paralyzed, the skin looked blue, and there was a blueness under the finger nails, as though the blood were imperfectly arterialized; the pulse was natural. The effect of the Cannabis was at its height in about three, and passed off in about five hours. The arm was in a powerless condition for half an hour, but friction would partially restore it.

Dr. Merret, of Detroit, in a short article on this subject, (*Medical Independent*, Sept. 1857,) describes the Cannabis as a deliriant which produces on many of the native population of the East, who use it freely, a most undesirable, in fact, a dangerous frame of mind. These evils are, however, fortunately counterbalanced by the antispasmodic effect of the drug. Dr. Merret speaks from a personal observation, during three years in a military hospital at Calcutta, of its administration in *tetanus*. In idiopathic cases it was most generally successful, and even in traumatic cases it showed its superiority over all other remedies. He mentions two cases of its successful use in England. Its relaxant effect has been proved to be an aid to taxis, in the reduction of hernia.

For farther details of the effect of doses of different degrees of strength, and the descriptions of the manner in which haschisch affects different individuals we would refer the reader to an article in the "National Review," copied into *Littell's Living Age*, Feb. 20th, 1858.

*Pæonia Officinalis—Peony.*—Dr. Abraham Livezey, of Lambertville, Penn., in "Observations on Convulsions of Children," (*Bost. Med. and Surg. Journ.*, Jan. 1857,) speaks of this plant possessing "peculiar anodyne or strong nervine powers; from the fact, farther, that it will allay the nervous twitches, the *subsultus*, or the peculiar startings of infants during repose." The mode of preparing and administering it is as follows:—Take of the dried root of Peony, grated, half a teaspoonful, scald it, and sweeten, and give the whole at once to a child 3 or 5 years old, three times a day. To an infant the same quantity may be given, in divided doses, during the day, or 24 hours. Dr. Levezey relates a case in which three physicians had failed to stop convulsions of daily recurrence during a period of several weeks, in a child 9 years old. Aunt Charity came along with dried "*piney*" root in her pocket, one dose of which she administered as above, and wonderful as it may appear, the convulsions ceased from that time. Other cases of a similar termination, by the same treatment, have come under Dr. Livezey's notice.

*Lobelia in Hydrocephalus.*—Dr. Ariel Hunton, of Vermont, in "Remarks on Hydrocephalus and Indigenous Plants," (*New Hampshire Journ. of Med.*, Jan. 1857,) points out the resemblance between hydrocephalus and the third stage of cholera infantum. The former is rather a misnomer in the present case, for in the stages which he thinks curable, Dr. H. does not admit that effusion has taken place. He believes that he has to deal with a case of arachnitis. His remedy for the state of things referred to, is the saturated Tincture of Lobelia; to a quart of this he adds half an ounce of the *Sanguinaria Canadensis*. Of this compound tincture he gives a teaspoonful to a child of two years, and a tablespoonful to an adult. He looks upon Lobelia as a direct sedative, which will, if taken without dilution, and without some stimulus conjoined with it, distress unduly the stomach, and give rise to other untoward symptoms. He considers it as an emetic; and, as such, he believes that, if properly administered, it is as safe as any other emetic—ipecacuanha not excepted. His treatment of arachnitis consists chiefly in the use of the Lobelia as an emetic every other day, and on the intermediate day, "a cathartic of calomel and a trifle of jalap."

*Indigenous Medicinal Plants.*—Dr. Hunton tells us that, for some years, he has made these plants the subject of his diligent study, in the daily use of them, and finds them, to say the least, as efficacious as foreign drugs. "*Scutellaria Lateriflora* and *Clematis Virginiana* are preferable, as nervines, to valerian. Among the abundance of our narcotics, I use (continues Dr. Hunton) more of the extract of stramonium than of all others, except opium. I prepare most of my narcotics with my own hand, to insure a pure article. If the practitioner does not choose to prepare these remedies himself, he may purchase them prepared by Tilden & Co., and will be sure of their purity."

*Asclepias Tuberosa.—Pleurisy Root.—Butterfly Weed.*—In the uncertainty which must be felt by every classifier as to the place which should be assigned to this plant—considering its diaphoretic, expectorant, cathartic, and tonic operation—we have put it under the head of Sedatives, looking to its ultimate soothing effect, rather than to its increasing, in various degrees, the activity of the functions, by which this result is reached.

Dr. Goodrake, of Clinton, Illinois, has made a "Report on the Medicinal Properties of the *Asclepias Tuberosa*," which finds its place in the "Transactions of the Illinois State Medical Society, 1857."

Dr. Goodrake gives the opinions of most of our systematic writers on the Materia Medica, respecting the medicinal properties of the Pleurisy root—and adds his own, which we believe to be the correct one, viz.: that in ordinary doses it is slightly sedative and astringent. Among the diseases to which this plant is well adapted, he enumerates catarrh, pneumonia, pleurisy, measles, incipient consumption, dysentery, cholera infantum, and the ephemeral fever, which is so common in children during the winter season. Dr. Goodrake has "given it in a variety of diseases, with a view to its properties as a diaphoretic, sometimes as an expectorant, and, in a great many cases, to quiet the bowels." He compares its action on the system to that of Dover's powder, but without the drawbacks attending this latter, of cerebral disturbance and impeded secretion from the kidneys. He lays particular stress on its remedial powers in both the diarrhœa and dysentery of young children. The root is given in powder, decoction, and extract. The preparation used by Dr. Goodrake in one case, was a decoction of the root, made by adding one ounce to a quart of boiling water, and keeping the fluid at a boil for an hour. Of this half a teaspoonful was taken every two hours in a case of dysentery intractable to the usual remedies. In another case, two drachms of the powdered root were combined with twelve grains of sugar of lead and six grains of camphor, the whole divided into six powders, of which one was to be taken every three hours; the patient drinking freely, the while, of pleurisy root tea. In a case of protracted infantile diarrhœa, Dr. Goodrake gave a decoction of the pleurisy root in milk—two drachms of the former to half a pint of the latter. Dose—a teaspoonful every hour. The improvement in the condition of the little patient after beginning this treatment was surprisingly rapid. "It may be well to remark, (says the writer,) that I allowed strong beef broth, well salted, during the whole of my treatment."

*Tartrate of Antimony in Colic.*—They who think of this antimonial salt only as an emetic and nauseant, have very limited and imperfect ideas of its therapeutical value, and of its activity as a sedative, or, to use the language of some Italian pathologists, as a counter or contra-stimulant, adapted to a large number of the phlegmasiæ, and producing its benign effect without nausea or any evacuation whatever. The annals of European medicine, especially of Italy and France, and, although in a more limited degree, of Ireland, prove this question beyond all doubt. Hence we can feel less surprise at the good effects of this substance in relieving colic, particularly of the stercoraceous variety, when administered in a state of proper dilution, as an enema.

Dr. C. Puffer, of Shelbourne Falls, Mass., gives, in the *Bost. Med. and Surg.*

*Journ.*, May, 1857, an account of a case of obstinate colic, which had resisted the usual remedies, but which yielded, in about forty minutes, to an enema, consisting of three grains of antimony in eight ounces of sweetened water.

Dr. Hosack, the eminent father of the present physician of New York, had recourse many years ago to this mode of treating a case of obstinate constipation and colic, with entire success.

*Cicuta in Sick-Headache.*—Dr. J. C. Norton, of Homer, Minnesota, in a Report on the Botany of Winona County, says of the Cicuta:—"In small doses, it is, according to my experience, a sure cure for nervous and sick-headache."

*Strychnia in Sick-Headache.*—D. J. B. McCaw, of Richmond, Va., in a paper on Sick-headache, (*Virg. Med. Journ.*, Jan. 1857,) expresses his conviction, as the results of some experiments made with the extracts both of *Ignatia amara* and of the *nux vomica* in old cases of long standing and great obstinacy, that in some of them at least, much benefit may be derived from some of the preparations of strychnia in diminishing the number of paroxysms and the susceptibility of the brain to future attacks.

*Belladonna in Arresting the Secretion of Milk in Swollen and Painful Breasts.*—Dr. S. Willey, of St. Paul, Minnesota, makes a very favorable statement (*Cincinnati Med. Obs.*) of the use of belladonna under the circumstances just named. He says:—"Upon reading Dr. Goulden's article in my number of the *London Lancet* for October, I resolved to make early trials of the remedy, and five cases had been furnished me of tumefied and painful breasts, not differing in any essential features from those reported by Dr. G., in which the free use of the extract of belladonna to the areola has acted like a specific in every instance. Once only did assistance seem required—citrate of magnesia having been given where protracted constipation obtained." Notes of two cases are furnished. In the first, distended and painful breasts followed weaning of the child, aged 11 months. Dr. Willey "ordered that the breasts should be well drawn, and an ointment composed of extract of belladonna and simple cerate, two-thirds of the former to one of the latter, to be rubbed upon the nipples and areola every two hours. Three hours subsequently, found breast flaccid and free from pain, much to the expressed wonder of the lady and the concealed surprise of myself. No refilling." The second case was that of a lady aged 21 years, who lost her child, aged seven months. "The application of belladonna *to the entire breasts* was made in this case, and complete relief was obtained in six hours. Extreme dilatation of the pupils, with strabismus, was produced, which passed away soon after the administration of paregoric in hot brandy sling. This was the only case where other than the nipples and areola were anointed."

(*To be continued in the November Number.*)

